# Metabolomic and immune alterations in long COVID patients with chronic fatigue syndrome

**DOI:** 10.3389/fimmu.2024.1341843

**Published:** 2024-01-18

**Authors:** Suguru Saito, Shima Shahbaz, Xian Luo, Mohammed Osman, Desiree Redmond, Jan Willem Cohen Tervaert, Liang Li, Shokrollah Elahi

**Affiliations:** ^1^ School of Dentistry, Division of Foundational Sciences, Edmonton, AB, Canada; ^2^ The Metabolomics Innovation Centre, University of Alberta, Edmonton, AB, Canada; ^3^ Department of Medicine, Division of Rheumatology, Edmonton, AB, Canada; ^4^ Department of Chemistry, University of Alberta, Edmonton, AB, Canada; ^5^ Li Ka Shing Institute of Virology, Faculty of Medicine and Dentistry, University of Alberta, Edmonton, AB, Canada

**Keywords:** sarcosine, serine, soluble CD14, depression, cognitive performance

## Abstract

**Introduction:**

A group of SARS-CoV-2 infected individuals present lingering symptoms, defined as long COVID (LC), that may last months or years post the onset of acute disease. A portion of LC patients have symptoms similar to myalgic encephalomyelitis or chronic fatigue syndrome (ME/CFS), which results in a substantial reduction in their quality of life. A better understanding of the pathophysiology of LC, in particular, ME/CFS is urgently needed.

**Methods:**

We identified and studied metabolites and soluble biomarkers in plasma from LC individuals mainly exhibiting ME/CFS compared to age-sex-matched recovered individuals (R) without LC, acute COVID-19 patients (A), and to SARS-CoV-2 unexposed healthy individuals (HC).

**Results:**

Through these analyses, we identified alterations in several metabolomic pathways in LC vs other groups. Plasma metabolomics analysis showed that LC differed from the R and HC groups. Of note, the R group also exhibited a different metabolomic profile than HC. Moreover, we observed a significant elevation in the plasma pro-inflammatory biomarkers (e.g. IL-1α, IL-6, TNF-α, Flt-1, and sCD14) but the reduction in ATP in LC patients. Our results demonstrate that LC patients exhibit persistent metabolomic abnormalities 12 months after the acute COVID-19 disease. Of note, such metabolomic alterations can be observed in the R group 12 months after the acute disease. Hence, the metabolomic recovery period for infected individuals with SARS-CoV-2 might be long-lasting. In particular, we found a significant reduction in sarcosine and serine concentrations in LC patients, which was inversely correlated with depression, anxiety, and cognitive dysfunction scores.

**Conclusion:**

Our study findings provide a comprehensive metabolomic knowledge base and other soluble biomarkers for a better understanding of the pathophysiology of LC and suggests sarcosine and serine supplementations might have potential therapeutic implications in LC patients. Finally, our study reveals that LC disproportionally affects females more than males, as evidenced by nearly 70% of our LC patients being female.

## Introduction

SARS-CoV-2 infection can result in asymptomatic or symptomatic presentations, ranging from mild to fatal coronavirus disease 2019 (COVID-19) (1). The coordinated action of the innate and adaptive immune systems provides adequate control of SARS-CoV-2 replication, resulting in the recovery of most immunocompetent individuals.

However, a substantial number of patients recovering from COVID-19 have reported a wide range of symptoms that last for many months or years after the onset of acute infection (2, 3). This syndrome is defined as post-acute COVID-19 syndrome (PACS), long-term COVID or “long hauler” patients (2, 4, 5). Multiple studies have evaluated LC symptoms in hospitalized patients, inevitably including only the more severe end of the spectrum (6–8). LC is not restricted to hospitalized patients and its estimated incidence is reported to lie between 10% and 60%, which depends on the definition of LC used and patient cohorts under study (9). LC is composed of heterogeneous sequelae that often affect multiple organ systems, with an impact on functional status and ability to work. The most prominent reported LC manifestations include breathlessness, headache, cough, chest pain, abdominal pain, muscle pain, fatigue, sleep disturbance, cognitive dysfunction (also termed “brain fog”), anxiety, and diarrhea (4, 9). Recent studies and surveys conducted by patient groups indicate that 50 to 80% of patients continue to have bothersome symptoms three months after the onset of COVID-19 disease even after tests no longer detect the virus in their body (7, 10, 11). In support of these observations, a recent study reported that 36% of COVID-19 patients experience several symptoms more than 3-6 months after the acute phase of the disease (3). Nevertheless, around 10% of individuals who have recovered may experience symptoms for over a year following the initial SARS-CoV-2 infection. These symptoms closely resemble those of myalgic encephalomyelitis or chronic fatigue syndrome (ME/CFS) (7, 12, 13), and/or exhibit other manifestations similar to systemic autoimmune rheumatic diseases (SARDs).

Any acute infection that damages multiple organs (e.g. cardiac, pulmonary, and/or renal involvement), like SARS-CoV-2, can be associated with lingering symptoms. In some individuals with persistent, debilitating fatigue following SRAS-CoV-2 infection, documented damage of vital organs may be a sufficient explanation for their fatigue. However, many cases of post-infectious fatigue follow acute infections that did not cause discernible organ damage or in those who had mild disease.

In people with lingering severe fatigue post-COVID-19 and without chronic cardiac, pulmonary, or renal dysfunction one likely explanation for the chronic fatigue is a state of chronic low-grade neuroinflammation (14). SARS-CoV-2 may form reservoirs resulting in viral-associated damage affecting the brain (15, 16), intestine, and liver, which can result in ongoing damage (17). It is known that SARS-CoV-2 enters the olfactory mucosa and can penetrate into the brain from the cribriform plate or via vagal pathways (18). Alternatively, the virus may also directly translocate across the blood-brain barrier (BBB) as a result of increased permeability stemming from inflammatory cytokines or inflammatory cells (e.g. monocytes) (19). Also, SARS-CoV-2 can reach neural tissue via circumventricular organs (CVOs) (20). Therefore, long-term neuropsychiatric symptoms may stem from chronic neuroinflammation and hypoxic injury (20). Indeed, recent studies have suggested that patients with LC may suffer from chronic hypoxic changes affecting the brain (21). Consistent with this, a vascular inflammation associated with hypoxia-inducible factor-1 is reported to contribute to neurological and cardiometabolic dysfunction in LC (22).

Another possible mechanism to explain LC may be that the initial infection triggers a broad immune response characterized by inflammation and immune dysregulation (1, 23). Furthermore, inflammation outside the brain can activate both immunological and somatic signals via both humoral and retrograde neuronal signals which largely involve the vagus nerve (24). These changes can culminate in symptoms of fatigue through the action of various cytokines which act on a “fatigue nucleus” or a collection of neurons dedicated to inhibiting energy-consuming activities that promote focusing on available energy stores for healing (25). Finally, impaired energy production associated with oxidative stress, glycolytic T cell metabolism, and immune alterations are suggested to play important roles in patients with idiopathic ME/CFS (26–29).

Given the global burden of LC and lack of understanding regarding its immunobiology, and pathophysiology, we aimed to systematically follow a cohort of individuals for > 12 months after the acute SARS-CoV-2 infection. This cohort was characterized by multiple clinical visits and laboratory analyses, and compared with a cohort of individuals who had been infected with SARS-CoV-2 but recovered without displaying any clinical symptoms. These individuals were recruited to the Long-COVID clinic at the University of Alberta hospital, with the recruitment was facilitated through patient community groups and the Alberta Long-COVID Facebook community. We also compared these two groups with a cohort of healthy controls (HCs, unexposed to SARS-CoV-2), and with patients suffering from a severe form of acute COVID-19 (A) who were admitted to the Intensive Care Unit (ICU). Collectively, these studies allowed us to analyze metabolomic profiles in these cohorts.

## Results

### Cohort characteristics

**Figure 1:**
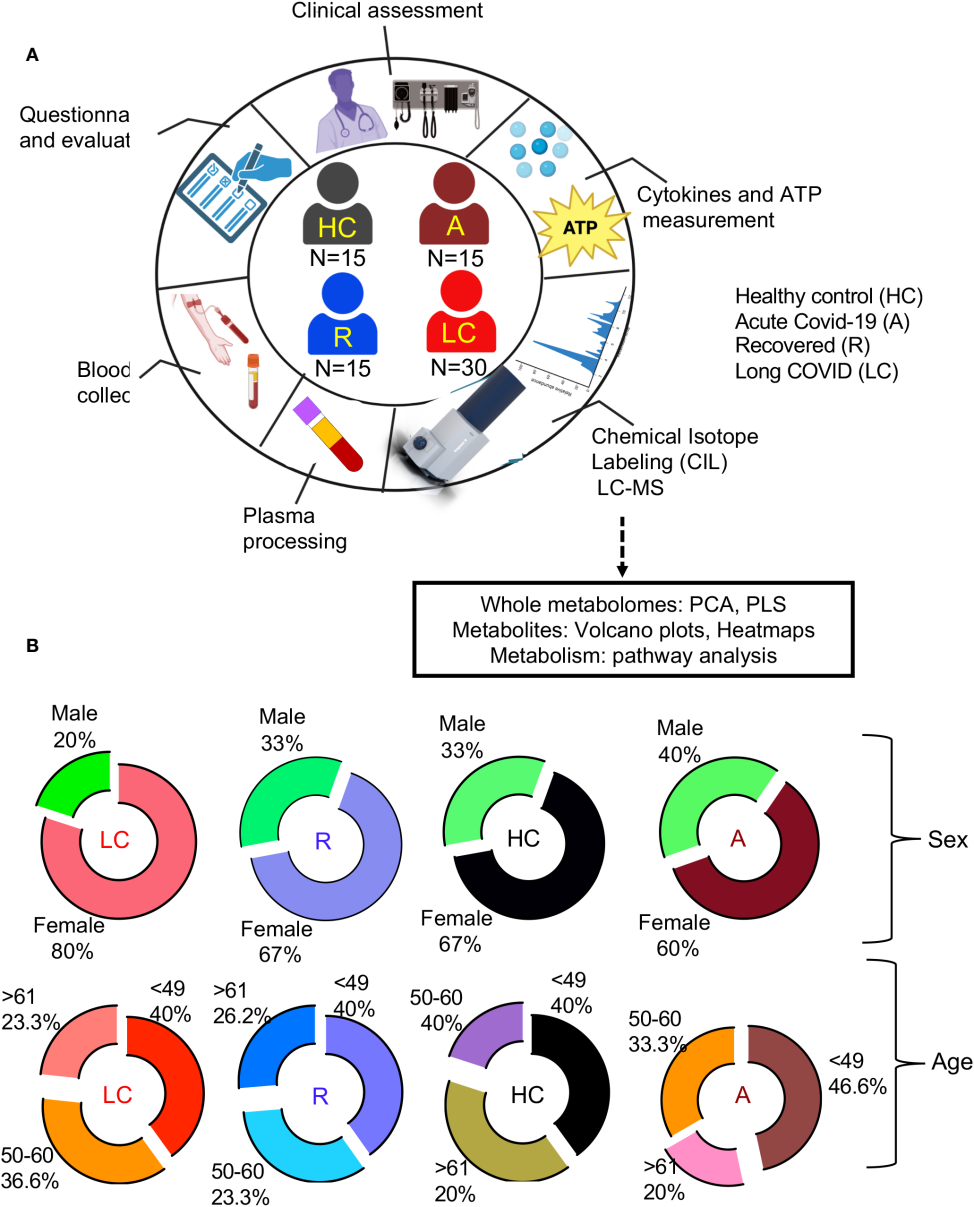
Demographic analysis of LC cohorts. **(A)** schematic of the study design. Numbers in center of diagram indicate participants in each study cohort (HC, healthy controls with no prior-SARS-CoV-2 exposure/vaccination; A, acute SARS-CoV-2 infected and ICU-admitted; LC, Long COVID; R, recovered). Outer ring indicates different studies/assays performed on patients/samples. **(B)** Demographic characteristics for each group displayed as ring charts for sex and age.

This is a single-centered cohort and consisted of 60 PCR-confirmed SARS-CoV-2 infected individuals (30 LC patients, 15 individuals without LC symptoms who were previously infected with SARS-CoV-2 but recovered without any symptoms per se (recovered group, denoted R), 15 acute hospitalized COVID-19 patients (A)) as well as 15 healthy controls (HCs) without acute symptoms and negative SARS-CoV-2 immunoassays (**Figure 1A**, **Supplementary Table 1**). Study participants were age -and sex-matched (**Figure 1B**) and all were infected with the Wuhan strain and recruited at ~12 months post the onset of acute infection (**Supplementary Table 1**). Considering that the majority of our LC and R participants had a mild acute infection, confounding health conditions were not common (**Supplementary Table 1**).

HCs were HIV, HCV, and HB seronegative, and mainly recruited pre-COVID-19 pandemic.

A key strength of this study was our unique focus on LC patients who met classification criteria for ME/CFS, which was not rigorously performed in any previous study. Those LC patients who did not fulfill ME/CFS criteria were excluded. Our LC patients were evaluated based on diagnostic criteria developed by CDC and WHO (30, 31) for ME/CFS. Moreover, we used a set of validated clinical questionnaires to capture the severity of symptoms for ME/CFS. This was conducted through in-person meetings using 7 different questionnaires including 1) De Paul Symptom Questionnaires (DSQ); 2) Functional Assessment of Chronic Illness Therapy (FACIT) Fatigue scale questionnaire; 3) FACIT-Dyspnea; 4) Fibromyalgia (FM) diagnostic criteria; 5) Cognitive Failure Questionnaire (CFQ); 6) The Pittsburgh Sleep Quality Index (PSQI): 7) The Hospital Anxiety and Depression Scale (HADS). We evaluated each patient based on different components of each questionnaire. For instance, the DSQ is used to assess symptoms related to ME/CFS (32). In this survey, patients were asked to rate 54 symptoms, and based on their responses we characterized them into 6 groups: fatigue (I), malaise (II), sleep difficulties (III), pain (IV), cognitive/neurological manifestations (V), and other (VI) which included autonomic, neuroendocrine, and immune manifestations. At the same time, the frequency of symptoms was evaluated using a 5-point Likert scale with 0: none of the time, 1: a little of the time, 2: about half the time, 3: most of the time, and 4: all of the time. Similarly, the severity of the symptoms was also recorded with 0: no symptom, 1: mild, 2: moderate, 3: severe, and 4: very severe. Each patient was considered positive if scored frequency and severity of ≥2 on at least one symptom from each category. For neurologic/cognitive manifestations, patients were considered positive if they scored a frequency and severity of ≥2 on at least two symptoms. For other categories (category VI), patients were considered positive if they had a minimum of one symptom from two of the autonomic, neuroendocrine, and immune manifestations. The diagnosis of ME/CFS was made if the patients met the criteria for categories I, II, III, IV, V, and VI (32–34).

A similar evaluation approach was utilized for the other questionnaires mentioned above. Thus, based on the obtained information from these questionnaires and clinical analyses, we characterized our LC patients.

### LC patients are presented with a distinct metabolomic profile compared to R, HC, and acute COVID-19 patients

**Figure 2:**
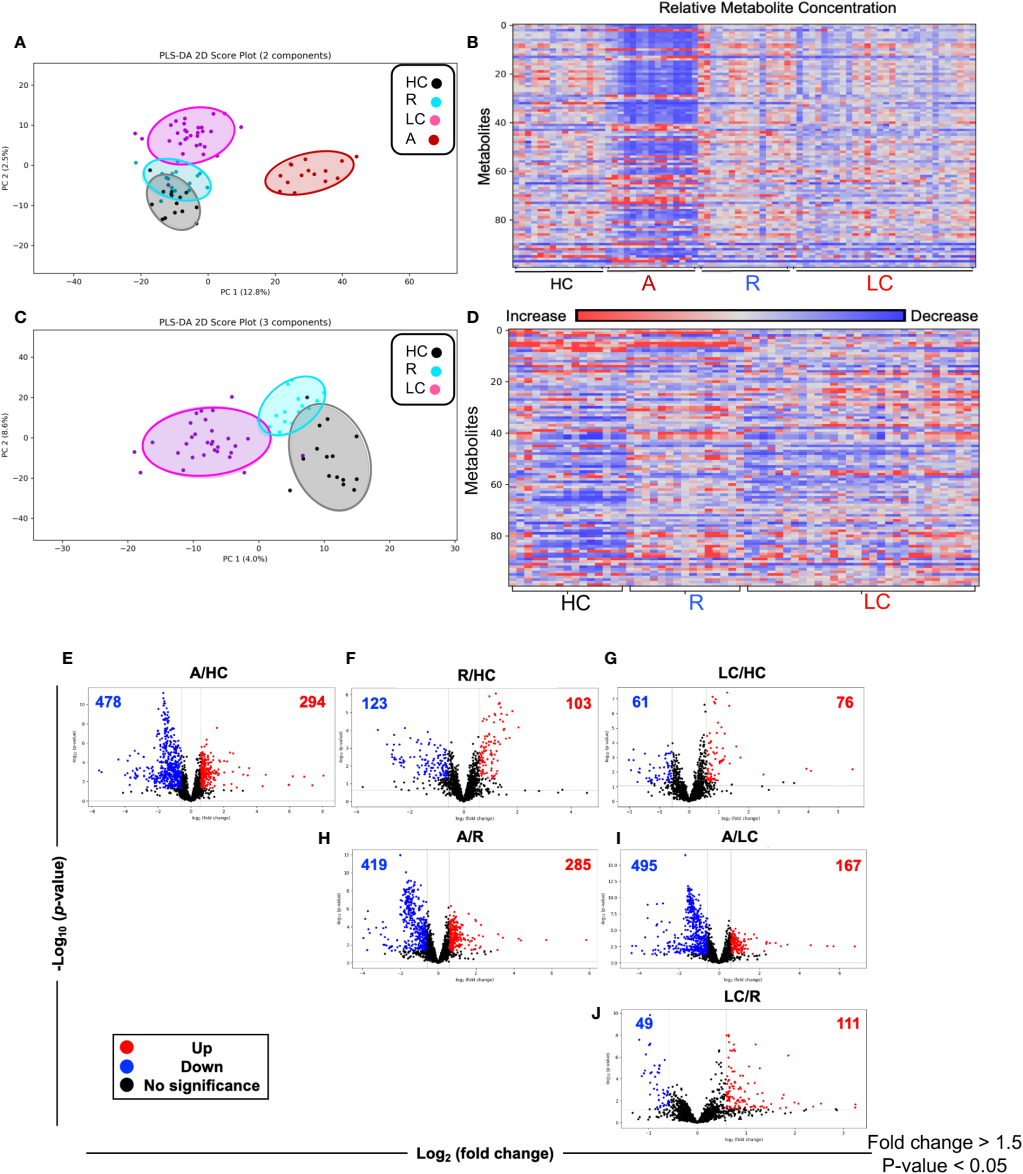
Altered metabolomic profile in SARS-CoV-2 infected individuals. **(A)** Partial least squares-discrimination analysis (PLS-DA) plot based on the metabolites in LC (n=30), acute COVID-19 (n=15), HC (n=15) and R (n=15). **(B)** Heatmap based on ANOVA using the top 100 significantly altered metabolites. The heatmap indicates the auto scaled levels of each metabolite in each sample, colored blue for decline and red for elevation as indicated on the horizontal bar. **(C)** Partial least squares-discrimination analysis (PLS-DA) plot based on the metabolites in LC (n=30), HC (n=15) and R (n=15). **(D)** Heatmap based on ANOVA using the 100 significantly altered metabolites. The heatmap indicates the auto scaled levels of each metabolite in each sample, colored blue for decline and red for elevation as indicated on the horizontal bar. **(E)** Volcano plots of significantly increased (red), decreased (blue) or unchanged (black) metabolites in acute A vs HCs. **(F)** Volcano plots of significantly increased, decreased or unchanged metabolites in R vs HC. **(G)** Volcano plots of significantly increased, decreased or unchanged metabolites in LC vs HC. **(H)** Volcano plots of significantly increased, decreased or unchanged metabolites in A vs R. **(I)** Volcano plots of significantly increased, decreased or unchanged metabolites in A vs LC. **(J)** Volcano plots of significantly increased, decreased or unchanged metabolites in LC vs R.

A total of 2584 metabolites were detected from 75 plasma samples including LC n=30, acute n=15, R n=15, and HC n=15. First, we compared the whole metabolome in our four study groups. Partial least squares-discrimination analysis (PLS-DA) and the heatmap showed a clear classification of the metabolomes between four groups (**Figures 2A, B**). The acute COVID-19 group showed clearly a distinct metabolome profile from the other three groups. Once the acute group was excluded from analysis, the LC group displayed a more distinct metabolome profile compared to the HC and R groups (**Figures 2C, D**).

While the LC and HC groups were separated, it appeared that the R group was located between these two groups (**Figure 2C**). Next, we characterized the difference at the single metabolite level between groups. Volcano plots showed 478 metabolites were significantly reduced while 294 metabolites were significantly increased in acute (A) COVID-19 patients compared to HCs (**Figure 2E**). Compared to HCs, 103 metabolites were elevated but 123 were declined in the R group (**Figure 2F**). Interestingly, the number of altered metabolites were much lower between LC and HC groups, showing 61 and 76 decreased and increased, respectively (**Figure 2G**). The number of altered metabolites between acute patients and the R group were very similar to those of acute/HCs with 419 increased and 285 decreased metabolites (**Figure 2H**). Compared to acute patients, we noted 495 metabolites were significantly reduced but 167 metabolites were elevated in the plasma of LC patients (**Figure 2I**). Finally, compared to the R group, LC patients had 49 reduced but 111 increased metabolites (**Figure 2J**). When we compared the top 100 altered metabolites between groups, we found that acute COVID-19 patients had a distinct metabolomic profile from HCs and R groups (**Supplementary Figures 1A, B**, **Supplementary Table 2**). Even though in general R individuals were segregated from HCs, three R patients assimilated with HCs (**Supplementary Figure 1C**, **Supplementary Table 2**). Next, we compared the pattern of metabolomic profile in LC patients versus the R and HC groups. These analyses indicated that when the top 100 altered metabolites were compared the LC group was segregated from both the R and HC groups (**Supplementary Figures 2A, B**, **Supplementary Table 2**). Overall, these observations demonstrate that LC and even R individuals present an altered metabolomic profile even ~12 months post the acute disease onset.

### Differential metabolic pathways in LC patients versus the R and HC groups

**Figure 3:**
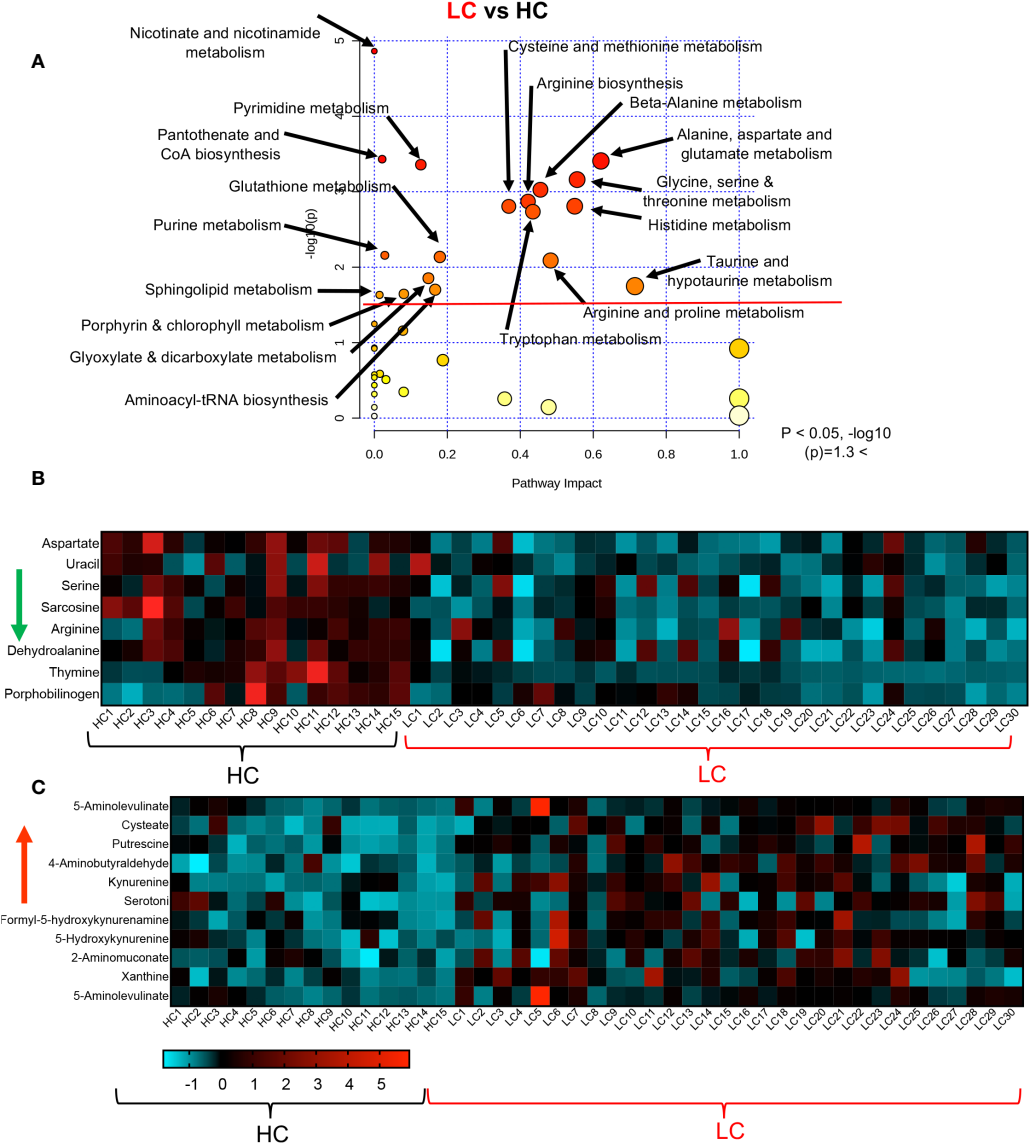
Altered metabolomic profile in LC vs HC. **(A)** Metabolic pathway enrichment analysis plot shows significantly altered pathways in LC vs HC. **(B)** The heatmap shows significantly decreased metabolites in different metabolic pathways in LC vs R. **(C)** The heatmap shows significantly elevated metabolites in different metabolomics pathways in LC vs R.

**Figure 4:**
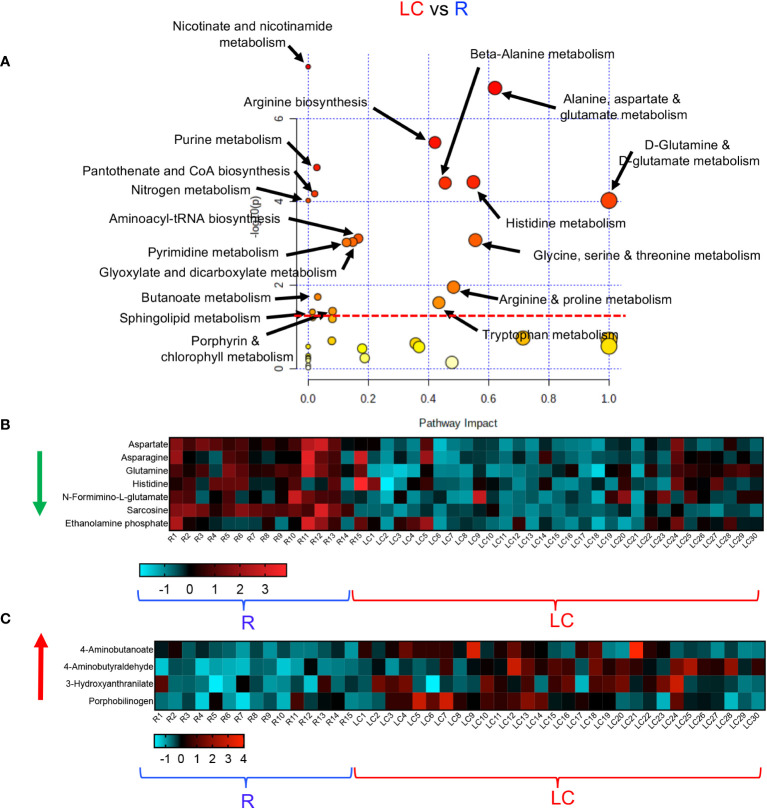
Altered metabolomic profile in LC vs R. **(A)** Metabolic pathway enrichment analysis plot shows significantly altered pathways in LC vs R. **(B)** The heatmap shows significantly decreased metabolites in different metabolic pathways in LC vs R. **(C)** The heatmap shows significantly elevated metabolites in different metabolomics pathways in LC vs R.

To obtain further insight into the differential metabolic characteristics of LC from R and HCs, we performed a metabolic pathway enrichment analysis (https://www.metaboanalyst.ca/) (35–37). Compared to HCs, these analyses revealed that 18 pathways were significantly altered in LC patients (**Figure 3A**). These alterations in metabolic pathways, resulted in a significant decline in the levels of aspartate, uracil, serine, sarcosine, arginine, dehydroalanine, thymine, and porphobilinogen in LC patients versus HCs (**Figure 3B**, **Supplementary Figure 3A**). However, the levels of other metabolites such as 5-Aminolevulinate, cysteate, putrescine, 4-Aminobutyraldehyde, kynurenine, serotonin, Formyl-5-hydroxykynurenamine, 5-Hydroxykynurenine, 2-Aminomuconate, xanthine, and 5-Aminolevulinate were significantly increased in LC patients (**Figure 3C**, **Supplementary Figure 3A**). Given the role of Metabolic pathway analyses between the LC and R group revealed also the alteration of 18 pathways, however, 15 out of these 18 pathways were similar to those observed between LC versus HCs (**Figure 4A**). The three differentially altered pathways between the LC vs HCs were glutamine, nitrogen, and butanoate mechanisms whereas cysteine and methionine, taurine, and glutathione metabolisms were altered between the LC and R groups (**Figure 4A**). Overall these alterations in metabolism pathways resulted in a substantial reduction in the levels of metabolites such as aspartate, asparagine, glutamine, histidine, N-formimino-glutamate, sarcosine, and ethanolamine phosphate in LC patients (**Figure 4B**, **Supplementary Figure 3B**). In contrast, metabolites such as 4-Aminobutanoate, 4-aminobutyraldehyde, 3-hydroxyanthranilate and porphobilinogen were elevated in the plasma of LC patients compared to the R group (**Figure 4C**, **Supplementary Figure 3B**).

**Figure 5:**
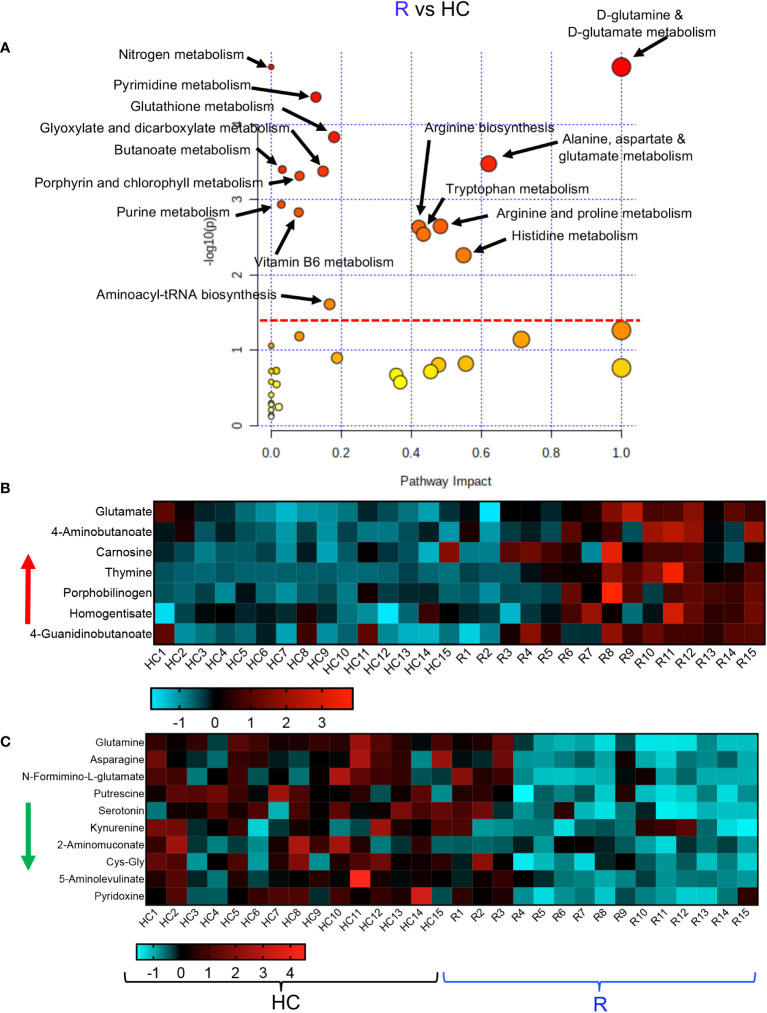
Altered metabolomic profile in R vs HC. **(A)** Metabolic pathway enrichment analysis plots of significantly altered pathways in the R vs HC group. **(B)** The heatmap shows significantly increased metabolites in different metabolic pathways in the R vs HC group. **(C)** The heatmap shows significantly decreased metabolites in different metabolic pathways in the R vs HC group.

Finally, we conducted a metabolic enrichment pathway analysis between the R and HCs. Although these individuals did not have any clinical symptoms and appeared to be healthy, they exhibited alterations in 15 metabolomics pathways compared with HCs (**Figure 5A**). Interestingly, 13 of these pathways were similar to those altered pathways between LC and HCs. Of note, the other two altered pathways in the R group were vitamin B and glutathione metabolisms (**Figure 5A**). These 15 altered metabolomics pathways resulted in increased levels of glutamate, 4-Aminobutanoate, carnosine, thymine, porphobilinogen, homogentisate and 4-Guanidinobutanoate in the R group (**Figure 5B**, **Supplementary Figure 4A**). At the same time, compared to HCs, the levels of a wide range of metabolites such as glutamine, asparagine, N-Formimino-glutamate, putrescine, serotonin, kynurenine, 2-Aminomuconate, Cys-Gly, 5-Aminolevulinate, and pyridoxine were reduced in the plasma of R group (**Figure 5C**, **Supplementary Figure 4A**). Despite the lack of clinical symptoms, the R group also exhibited a differential metabolomics profile compared to HCs. Taken together, these observations imply substantial metabolic pathways alterations in LC patients.

### Differential metabolomic profile of acute COVID-19 patients vs HCs

In agreement with previous reports (38), we observed alteration in plasma metabolite levels of acute patients vs HCs. Pathway analysis between acute and HCs revealed alteration in 29 pathways as indicated in **Supplementary Figure 4B**. These modifications in metabolism pathways resulted in a substantial increase in the levels of 48 metabolites such as Xanthine, ascorbate, Cys-Gly, kynurenine, phenylalanine, 5-hydroxykynurenine, and others as outlined in **Supplementary Figures 5A, B**. In contrast, we found that these metabolomic alterations led to a reduction in the plasma level of different metabolites including uridine, thymine, carnosine, theophylline, aspartate, taurine, serine, glutamate, tryptophan, arginine, and 53 other metabolites in the acute patients compared to HCs (**Supplementary Figures 6A, B**).

### Differential metabolomic profile of acute COVID-19 patients vs R individuals

We also compared a metabolomic profile of those recovered from COVID-19 infection with the acute stage of the disease. Pathway analysis between acute and R indicated alteration in 36 pathways as indicated in **Supplementary Figure 7**. These modifications in metabolism pathways resulted in a substantial increase in the levels of 39 metabolites in acute COVID 19 patients compared to the R group such as phenylaniline, pyrimidodiazepine, xanthine, ascorbate, kynurenine, 5-hydroxykynurenine and others as outlined in **Supplementary Figures 8A, B**. In contrast, we found that these metabolomic alterations led to a reduction in the plasma level of 66 different metabolites in acute patients versus the R group (**Supplementary Figures 9A-C**). Finally, we compared the metabolomic profile of LC vs acute COVID-19 patients. These analyses revealed alteration in 30 metabolism pathways (**Supplementary Figure 10**). These changes in metabolism pathways were associated with a significant increase in 29 metabolites as shown in **Supplementary Figure 11**. In contrast, we observed a significant reduction in 16 metabolites in LC patients compared to those with acute COVID-19 disease (**Supplementary Figure 12**).

### Increased levels of pro-inflammatory cytokines and auto-antibody in LC

**Figure 6:**
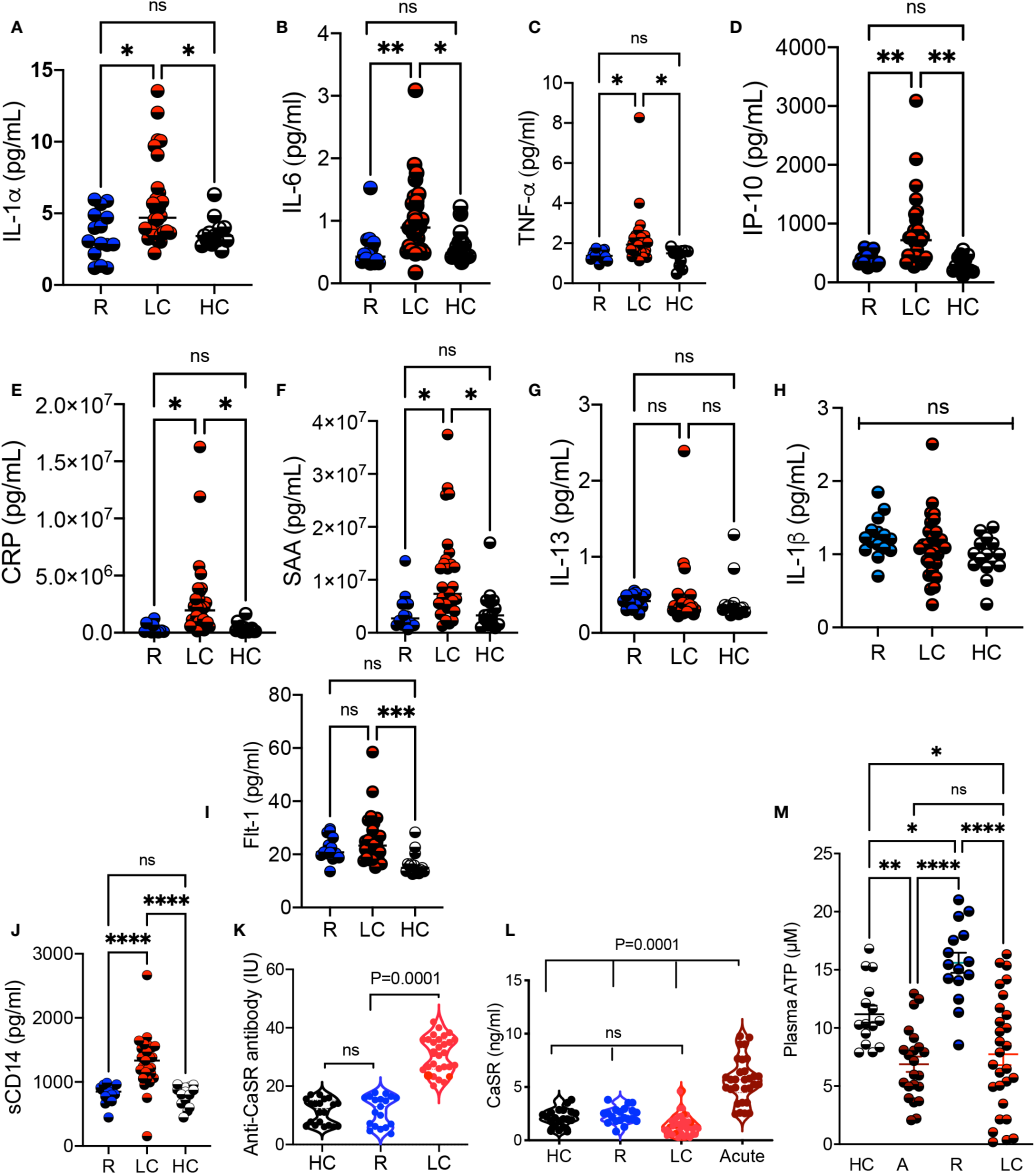
Elevated levels of proinflammatory biomarkers in LC patients. **(A)** Cumulative data comparing the plasma IL-1α, **(B)** IL-6, **(C)** TNF-α, **(D)** IP-10, **(E)** CRP, **(F)** SAA, **(G)** IL-13, and **(H)** IL-1β, **(I)** Flt-1, and **(J)** soluble CD14 measured by ELISA in the plasma of R, LC, and HC groups. **(K)** Comparing the Anti-CaSR antibody levels in plasma samples of HC, R, and LC group. **(L)** Soluble CaSR levels in plasma samples of HC, R, LC and acute COVID-19 patients. **(M)** Cumulative data of the plasma ATP in HC, acute, R, and LC groups. Kruskal–Wallis analysis with Dunn’s multiple comparisons test. ns, not significant. Each dot represents a study subject. * p < 0.5, ** p ≤ 0.01, *** p ≤ 0.001, and **** p ≤ 0.0001.

Considering the alteration in a variety of pathways such as tryptophan-kynurenine and Xanthine/hypoxanthine, we quantified the levels of inflammatory cytokines. We found a significant increase in the plasma IL-1α, IL-6, TNF-α, IP-10, and liver-associated active phase proteins (CRP and SAA) in LC patients versus R and HCs (**Figures 6A-F**). However, the plasma IL-13 and IL-1β concentrations remained unchanged between groups (**Figures 6G, H**). Although we did not find any significant difference between soluble Flt-1, also known as vascular endothelial growth factor receptor 1, levels in the plasma of R and LC patients, LC group exhibits a significant plasma elevation compared to the HC cohort (**Figure 6I**). We also quantified sCD14, a nonspecific monocyte activation marker, levels in the plasma of our study participants, which showed a significant elevation in LC patients compared to the HC and R groups (**Figure 6J**). In light of hypocalcemia in COVID-19 patients and its association with hypoparathyroidism (39, 40), we detected significant levels of the anti-CaSR antibody (calcium receptor) in LC cohort (**Figure 6K**). This observation forced us to measure the soluble form of CaSR in our different cohorts. Interestingly, we observed the elevation of soluble/shed CaSR in the plasma of acute COVID-19 patients, which was not the case in LC patients (**Figure 6L**). Finally, considering the impact of COVID-19 infection on the purinergic system (41), we quantified the plasma levels of ATP in our different cohorts. We found a significant reduction in the plasma ATP levels in acute COVID-19 patients compared to HCs and R groups (**Figure 6M**). Notably, LC patients had a substantially reduced level of ATP in their plasmas compared to either R and HCs (**Figure 6M**). Intriguingly, the R groups exhibited a significant elevation in the plasma ATP compared to the HC and R groups (**Figure 6M**). Overall, these observations imply a dysregulated and inflammatory immune response in LC patients.

### The association of sarcosine and serine with clinical manifestations in LC

**Figure 7:**
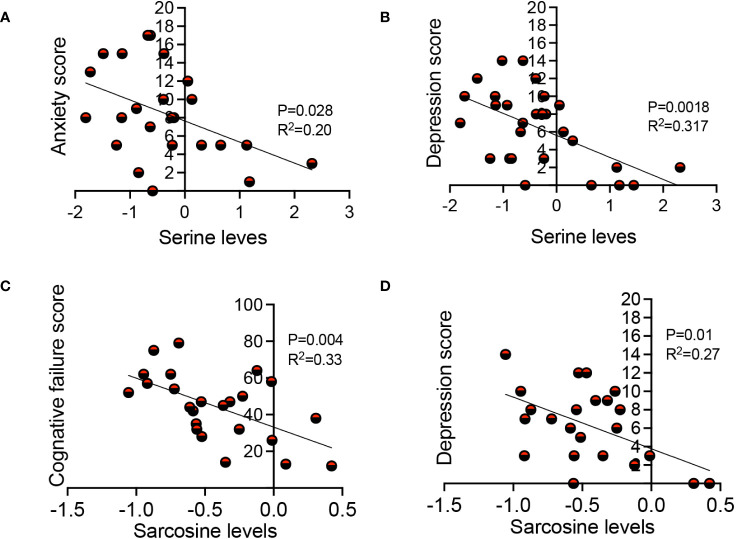
The association of sarcosine and serine with clinical symptoms in LC. **(A)** The correlation between serine levels with anxiety score, and **(B)** depression score in LC patients. **(C)** The correlation between sarcosine levels with cognitive failure score, and **(D)** depression score in LC patients. Each dot represents a study subject. P values and R^2^ were obtained by Simple linear repression analysis.

In our investigations of metabolite levels associated with symptoms commonly seen in LC patients such as cognitive function, anxiety, chronic pain, and depression in LC patients, we conducted additional analyses. Despite finding no association between serotonin, tryptophan, aspartate, and kynurenine plasma levels with these symptoms, interestingly, we observed a subtle inverse correlation between sarcosine concentrations and both cognitive failure function scores (**Figure 7A**) and depression scores in LC patients (**Figure 7B**). Moreover, our observations revealed a similar association between the plasma serine levels and anxiety and depression scores in LC patients (**Figures 7C, D**). These initial findings suggest a potential link between reduced levels of sarcosine and serine metabolites and some of the clinical symptoms seen in LC patients.

## Discussion

Diverse metabolomic alterations are reported in the acute phase of SARS-CoV-2 infection (42, 43). However, there have been limited studies to determine whether such metabolomic signature is transient or last for a while after the recovery from the acute disease. To elucidate potential mechanisms, the plasma metabolome of LC patients was investigated and compared to age-sex-matched acutely ill COVID-19, R, and HCs. Our metabolomic analysis revealed alterations in a variety of metabolic pathways in both LC and R individuals even 12 months after the onset of COVID-19 disease. Although our metabolomic analyses are descriptive, they provide insights into metabolomic changes following SARS-CoV-2 infection that lasts for months and possibly years in the absence of a detectable virus. Our results suggest that LC has the potential to severely impact the functionality of different organs. It is possible to speculate that the delay in the tissue repair process following acute COVID-19 disease and the inflammatory milieu result in metabolomic alterations in LC. For example, we noted a reduction in glutamine and ornithine plasma levels, which suggests a disturbance in amino acid and nitrogen metabolism as reported in CFS (44). This may also be consistent with impaired mitochondrial biogenesis as described in circulating lymphocytes in chronic inflammatory conditions (45) and in patients with idiopathic ME/CFS (29).

Also, we detected a reduction in serine concentration, which was inversely associated with depression and anxiety scores in LC patients. Considering that this amino acid is essential for central nervous system (CNS) function and development (46), its decline has been associated with severe neurological disorders (47, 48). Therefore, recognition of serine deficiency in LC is highly important because serine therapy has shown promising outcomes in neurological conditions (49) and in reducing depression and cognitive impairment (50).

We found a significant decline in plasma sarcosine levels in LC patients, which is also reported in critically ill COVID-19 patients (35). Sarcosine, a glycine transporter-I inhibitor, is reported to reduce depression-like symptoms in animal models and human subjects (51). Although, it is unclear whether sarcosine is the cause or the effect, our findings support a link between the low sarcosine levels with cognitive dysfunction and depression scores in LC patients. In support of this concept, sarcosine supplementation has shown promising outcomes in those with learning and memory deficits (52), Parkinson’s disease (53), and depression (54). Ultimately, whether sarcosine supplementation can be harnessed to target some of the deleterious manifestations of LC through stimulation of autophagy (55) is an intriguing potential therapy to explore further.

Despite previous reports on the role of tryptophan/serotonin in anxiety and depression (56), our results did not support the idea that changes in these metabolites are associated with depression, anxiety, and cognitive function scores in our LC patients.

The lower arginine in LC patients as reported in acute COVID-19 (57) can be related to increased frequency of CD71+ erythroid cells (CECs) expressing arginase-I and II (58, 59) or the pathology associated with the effects of the virus on the endothelium (60). Based on this, there is a possibility that arginine has beneficial effects on endothelium function in LC patients.

Other metabolites such as L/D-aspartate and serine amino acids are enriched in the brain and their reduction/dysfunction have been associated with neurological disorders (61). Considering the ability of D-aspartate to cross the BBB (62), D-aspartate supplementation may have therapeutic potential in LC patients. Similarly, arginine and aspartate intake has been associated with delayed muscle fatigue and increased exercise tolerance (63), which could be beneficial in LC with ME/CFS.

Notably, a lower ATP plasma level may imply the impact of LC on the glycolytic and mitochondria pathway for cellular energy production. Such alterations may result in dysfunction or symptoms involving high energy-consuming organs such as the brain, liver, heart, and skeletal muscles (64). While intermediate metabolites associated with TCA cycle (Tricarboxylic Acid Cycle) such as fumarate, succinate, and malate were not significantly different between the groups, we suggest a decrease in mitochondrial activity may result in lower ATP generation as reported in other inflammatory conditions (65, 66). Alternatively, the lingering low level of hypoxia (1) following the acute COVID-19 infection may also contribute to lower ATP levels. Overall, the elevation of pro-inflammatory cytokines may result in mitochondrial dysfunction and/or alteration in LC patients. In line with our observations, mitochondrial dysfunction has been reported in ME/CFS (67, 68). Although we were unable to quantify tissue-specific ATP levels, it is likely to suggest that a lower plasma ATP in LC may contribute to, at least in part, ME/CFS. Given the role of ATP in tissue repair, its low level in LC patients may delay the tissue repair process resulting in sustained inflammatory condition. Therefore, increased ATP levels by ATP regulators may provide a strategy for cell/tissue protection in LC as reported in neurological condition (69).

It has been shown that tryptophan metabolism is enhanced in COVID-19 infection, leading to a decrease in tryptophan and increased levels of kynurenine metabolites (43), which can have multiple deleterious effects on the musculoskeletal system (70). Therefore, increased plasma kynurenine could be linked to LC symptoms. Similarly, increased plasma serotonin levels may explain the mechanism associated with diarrhea in LC patients as reported in acute COVID-19 (71). Similarly, elevated Xanthine/hypoxanthine in LC patients can be associated with increased pro-inflammatory cytokine and acute phase proteins (e.g. IL-1, TNF- *α*, IL-6, CRP, and SAA) as reported elsewhere (72). While we did not specifically evaluate the M1/M2 macrophage phenotype in our study cohorts, the observed pro-inflammatory immune profile suggests an activation of M1-type macrophages in patients with LC. This finding contrasts with a recent study that proposed higher M2-type macrophage activity in LC (73). Several factors may account for the disparities in our results. Our LC and R groups were recruited 12 months post-acute SARS-CoV-2 infection, whereas Kovarik et al. conducted their study at a much shorter interval (3 months) (73). Additionally, our study cohorts were vaccine-naïve, while subjects in the other study had received 2 doses of SARS-CoV-2 vaccines. Lastly, our cohort represents a homogeneous subset of LC patients with ME/CFS. Other factors such as reduced thymine, increased Flt-1 and pro-inflammatory analytes may favor vascular inflammation in LC (22, 74). Moreover, elevated plasma levels of sCD14 (monocyte activation marker) support the residual general immune activation in LC.

Although we did not find any difference in glutathione and methionine pathways between LC and R groups, they were significantly altered in LC vs HCs. A recent study has reported the prevalence of the genotype CC of the methylenetetrahydrofolate reductase (*MTHFR*) gene in LC patients (75). Hence, this genetic polymorphism may explain differences in cysteine and methionine metabolism in LC patients.

Likewise, alteration in glutathione metabolism may promote oxidative stress and an inflammatory milieu in LC patients as reported in acute COVID-19 disease (76).

Taken together, our results demonstrate diverse metabolomic alterations in patients with LC syndrome. The existence of metabolomic alterations and chronic systemic inflammation even 12 months after acute SARS-CoV-2 infection suggests a dysregulated immune response. This has been supported by the presence of autoantibodies (77, 78). In light of hypocalcemia in COVID-19 patients and its association with hypoparathyroidism (39, 40), we detected significant levels of the anti-CaSR antibody in our LC cohort. Mechanistically, the consequences of SARS-CoV-2 infection-induced mediators (e.g. cytokines, chemokines, growth factors, and metabolites) can have a prominent impact on hematopoietic stem and progenitor cells (HSPCs)^1^. Lingering dysregulated hematopoiesis in LC patients suggests a potentially impaired antiviral response and/or increased innate immune response. Subsequently, an impaired antiviral response may increase antigen persistence and promote chronic inflammation which contributes to metabolomic alteration. Therefore, our studies add novel insights into a growing body of evidence implying that a combination of virus and host factors such as residual immune activation, hematopoietic dysregulation, and autoimmune phenomena could contribute to LC syndrome.

While a recent study has reported alterations in the taurine pathway in LC patients (79), our findings do not support this concept. We did not observe any changes in taurine and hypotaurine metabolism in LC compared to the R group. However, a slight alteration in this metabolic pathway was noted in LC when compared to HCs. In contrast, our results reveal a significant reduction in sarcosine and serine concentrations in LC patients compared to both HCs and the R group. Notably, the inverse correlation between sarcosine/serine levels and cognitive dysfunction/depression suggests that sarcosine/serine supplementations might have therapeutic applications in LC patients.

The substantial differences in our findings compared to the recent study (79) might be related to two main factors. Firstly, our study subjects were recruited 12 months post the onset of acute infection, as opposed to 6 months in the recent report. It is possible to speculate that alterations in the taurine pathway are transient and does not persist until 12 months post the acute disease onset. Secondly, our cohort comprised a homogenous subset of LC patients with ME/CFS, in contrast to the heterogenous cohort in the recent study. In summary, our study provides an insight into lingering immune activation and altered/impaired mitochondrial bioenergetics in LC patients with ME/CFS. In particular, our global metabolomic analyses revealed important therapeutic targets in LC patients. Nevertheless, it’s important to note that further research and clinical trials are needed to validate and establish the efficacy of sarcosine and serine supplementations for therapeutic purposes in LC.

We are aware of several study limitations. This is a single-centered study, therefore, similar studies in larger cohorts of LC patients with ME/CFS are needed. Also, we were unable to collect blood samples on multiple occasions to determine changes in metabolites over time. This study was done on patients infected with the Wuhan strain of SARS-CoV-2. In lights of differential immune response in those infected with other SARS-CoV-2 viral variants (e.g. Delta, Omicron, etc.) (80), further comparative studies are needed. Although sex is a crucial biological variable, the limited representation of males with LC compared to females in our cohort prevented us from observing any significant differences. Moreover, we encountered difficulties in recruiting an equal number of study subjects for each group due to participants availability. This imbalance in group sizes has implications for the statistical power of our study. Our metabolomic analyses did not include lipids; therefore, the lack of data on lipid metabolism in LC prevents our study from providing a comprehensive picture of metabolic alterations. This limitation highlights the importance of future studies incorporating lipid analysis in large prospective LC cohorts. Finally, further studies in animal models of Long-COVID may provide deeper insight into disease pathogenesis and validation of our observations for therapeutic purposes.

## Materials and methods

### Study population

Sixty PCR-confirmed SARS-CoV-2 infected individuals comprised of 30 LC patients, 15 individuals without LC symptoms who were previously infected with SARS-CoV-2 but recovered without any symptoms per se (recovered group, denoted R), 15 acute hospitalized COVID-19 patients) as well as 15 healthy controls (HCs) without acute symptoms and negative SARS-CoV-2 immunoassays (**Figure 1A**, **Supplementary Table 1**). All individuals were infected with SARS-CoV-2 Wuhan viral strain in 2020, which was confirmed by PCR at University of Alberta Hospital, Edmonton. Both LC and R groups were recruited ~12 months after the onset of SARS-CoV-2 infection (**Supplementary Table 1**).

### Ethics statement

This study was approved by the Human Research Ethics Board (HREB) at the University of Alberta (protocol # Pro00099502). A written informed consent form was obtained from all participants but a waiver of consent was obtained for those admitted to the ICU.

### Plasma collection

Plasma samples were collected following centrifugation of fresh blood samples and kept frozen at -80 until use.

### Metabolomic profiling

Frozen plasma samples were thawed at RT, then were centrifuged at 600 g for 5 min. The 100 μl of soluble fraction was transferred into a new tube and mixed with 300 μl of LC-MS grade methanol. The samples were stored at -20°C for 30 min, then were centrifuged at 16,260 g to let precipitate proteins. The supernatants were transferred into new tubes and dried up completely. The dried samples were reconstituted by dissolving in 85 μl of water. The metabolomic analysis was performed by following the Chemical Isotope Labeling (CIL) LC-MS protocol described in our previous report (81). Briefly, each individual sample was labeled with ^12^C_2_-dansyl chloride. The pooled sample was generated by mixing each of the individual samples, and labeled by ^13^C_2_-dansyl chloride. After mixing each ^12^C-labeled sample with an equal volume of the ^13^C-labeled pool, the ^12^C-/^13^C- mixtures were injected onto LC-MS for analysis. The LC-MS system was Agilent 1290 LC linked to Brukler Impact II QTOF Mass spectrometer. The samples were injected into an Agilent Eclipse Plus reversed-phase C18 column (2.1 mm × 150 mm, 1.8 μm particle size, 95 Å pore size) for separation. Solvent A was 0.1% (v/v) formic acid in water, and solvent B was 0.1% (v/v) formic acid in acetonitrile. The chromatographic conditions were: *t*= 0 min, 25% B; *t*= 10 min, 99% B; *t*= 15 min, 99% B; *t*= 15.1 min, 25% B; *t*= 18 min, 25% B. The flow rate was 400 μL/min. All MS spectra were obtained in the positive ion mode. The MS conditions used for Q-TOF were as follows: nebulizer, 1.0 bar; dry temperature, 230°C; dry gas, 8 L/min; capillary voltage, 4500 V; end plate offset, 500 V; spectra rate, 1.0 Hz. The raw data were exported by Bruker Data Analysis 4.4. as.csv files, and the.csv files were processed by IsoMS Pro 1.2.15. The ^12^C/^13^C- peak pairs were extracted, and missing values in the data matrix were filled. Metabolite identification was performed by searching against libraries with different confidence levels. Tier 1 library (DnsID library) contains accurate mass, MS/MS, and retention time information; Tier 2 library (Linked ID library) contains accurate mass and predicted retention time information; Tier 3 library (My Compound ID library, www.MycompoundID.org) contains accurate mass of predicted metabolites. IsoMS Pro was also used for performing the univariate analysis (volcano plot) and multivariate analysis (PCA and PLS-DA). The data file containing Tier 1 metabolites was uploaded to Metaboanalyst 5.0 (www.metaboanalyst.ca) for generating Heatmap. Pathway enrichment analysis was performed by using Metaboanalyst 5.0.

### Cytokine and chemokine multiplex analysis

Frozen plasma samples at -80 °CC were thawed and centrifuged for 15 min at 1500g followed by dilution for quantifying cytokine and chemokine profiles, respectively. The concentration of cytokines and chemokines was quantified using the V-PLEX from Meso Scale Discovery (MSD). Data were acquired on the V-plex^®^ Sector Imager 2400 plate reader. Analyte concentrations were extrapolated from a standard curve calculated using a four-parameter logistic fit using MSD Workbench 3.0 software according to reported protocols (82–84).

The plasma concentration of ATP was quantified using the ATPlite luminescence assay system (PerkinElmer, MA) as we have reported elsewhere (85). The plasma was subjected to ELISA kit sCD14 (R&D, 383CD-050) (80) and Anti-CaSR (EAGLE Biosciences).

### Statistical analysis

We initially determined the distribution of data using the Wilks-Shapiro test and then based on the distribution of data the appropriate test was used. When data were not normally distributed the non-parametric tests such as the Mann-Whitney U-test or Kruskal–Wallis analysis of variance were used. Depending on the data set different *post hoc* tests were used. The Dunn’s multiple comparisons as the *post hoc* test for the Kruskal–Wallis analysis. The Tukey-Kramer test was used for one-way ANOVA with multiple comparisons. For nonparametric correlational studies tow-tailed Spearman correlation was used. *P*-values are shown in the graphs and measures are expressed as mean ± SEM and *P-*value < 0.05 was considered to be statistically significant. In violin plots, the middle line represents median, the bottom line 1^st^ quartile, and to top line 3^rd^ quartile. No randomization was performed and no data points were excluded.

## Data availability statement

The original contributions presented in the study are included in the article/**Supplementary Material**. Further inquiries can be directed to the corresponding author.

## Ethics statement

The studies involving humans were approved by Ethics Board of the University of Alberta. The studies were conducted in accordance with the local legislation and institutional requirements. The participants provided their written informed consent to participate in this study.

## Author contributions

SSa: Conceptualization, Writing – review & editing, Data curation, Formal Analysis, Investigation, Methodology, Software, Validation. SSh: Data curation, Formal Analysis, Investigation, Methodology, Writing – review & editing, Visualization. XL: Data curation, Formal Analysis, Investigation, Writing – review & editing, Software. MO: Investigation, Conceptualization, Resources, Writing – review & editing. DR: Investigation, Writing – review & editing, Data curation. JC: Writing – review & editing, Resources. LL: Resources, Writing – review & editing, Software. SE: Resources, Writing – review & editing, Conceptualization, Funding acquisition, Project administration, Supervision, Writing – original draft.

## Supplementary material

Supplementary Figure 1
**(A)** The heatmap of the top 100 altered metabolites in acute COVID-19 patients (A) vs HC. **(B)** A vs R, and **(C)** HC vs R.

Supplementary Figure 2
**(A)** The heatmap of the top 100 altered metabolites in R vs LC, and **(B)**, LC vs HC.

Supplementary Figure 3
**(A)** Cumulative data showing relative concentrations of increased and decreased metabolites in the LC vs R group. **(B)** Cumulative data showing relative concentrations of increased and decreased metabolites in LC vs R. Each dot represents a study subject. *P* values were calculated using one-way ANOVA.

Supplementary Figure 4
**(A)** Cumulative data showing relative concentrations of increased and decreased metabolites in R vs HC. **(B)** Metabolic pathway enrichment analysis plot shows significantly altered pathways in A vs HC. Each dot represents a study subject. Data are analyzed by one-way ANOVA.

Supplementary Figure 5
**(A, B)** Cumulative data showing relative concentrations of increased metabolites in the A vs HC group. Each dot represents a study subject. *P* values were calculated using one-way ANOVA.

Supplementary Figure 6
**(A, B)** Cumulative data showing relative concentrations of decreased metabolites in the A vs HC group. Each dot represents a study subject. *P* values were calculated using one-way ANOVA.

Supplementary Figure 7 Metabolic pathway enrichment analysis plot shows significantly altered pathways in A vs R.

Supplementary Figure 8
**(A, B)** Cumulative data showing relative concentrations of decreased metabolites in the R vs A group. Each dot represents a study subject. *P* values were calculated using one-way ANOVA.

Supplementary Figure 9 Cumulative data showing relative concentrations of elevated metabolites in the R vs A group. Each dot represents a study subject. *P* values were calculated using one-way ANOVA.

Supplementary Figure 10 Metabolic pathway enrichment analysis plot shows significantly altered pathways in A vs LC.

## References

[1] Elahi S. Hematopoietic responses to SARS-CoV-2 infection. Cell Mol Life Sci CMLS. (2022) 79(3):187. doi: 10.1007/s00018-022-04220-6 35284964 PMC8918078

[2] Desai AD Lavelle M Boursiquot BC Wan EY. Long-term complications of COVID-19. Am J Physiol Cell Physiol (2022) 322(1):C1–C11. doi: 10.1152/ajpcell.00375.2021 34817268 PMC8721906

[3] Taquet M Dercon Q Luciano S Geddes JR Husain M Harrison PJ. Incidence, co-occurrence, and evolution of long-COVID features: A 6-month retrospective cohort study of 273,618 survivors of COVID-19. PloS Med (2021) 18(9):e1003773. doi: 10.1371/journal.pmed.1003773 34582441 PMC8478214

[4] Gorna R MacDermott N Rayner C O'Hara M Evans S Agyen L. Long COVID guidelines need to reflect lived experience. Lancet (2021) 397(10273):455–7. doi: 10.1016/S0140-6736(20)32705-7 PMC7755576 33357467

[5] Mehandru S Merad M. Pathological sequelae of long-haul COVID. Nat Immunol (2022) 23(2):194–202. doi: 10.1038/s41590-021-01104-y 35105985 PMC9127978

[6] Bakamutumaho B Cummings MJ Owor N Kayiwa J Namulondo J Byaruhanga T. Severe COVID-19 in Uganda across two epidemic phases: A prospective cohort study. Am J Trop Med Hyg (2021) 105(3):740–4. doi: 10.4269/ajtmh.21-0551 PMC8592357 34370701

[7] Couzin-Frankel J. The long haul. Science (2020) 369(6504):614–7. doi: 10.1126/science.369.6504.614 32764050

[8] Parums DV. Editorial: long COVID, or post-COVID syndrome, and the global impact on health care. Med Sci Monitor (2021) 27:e933446. doi: 10.12659/MSM.933446 PMC8194290 34092779

[9] Sapkota HR Nune A. Long COVID from rheumatology perspective - a narrative review. Clin Rheumatol (2022) 41(2):337–48. doi: 10.1007/s10067-021-06001-1 PMC8629735 34845562

[10] Carfi A Bernabei R Landi F Gemelli Against C-P-ACSG. Persistent symptoms in patients after acute COVID-19. Jama (2020) 324(6):603–5. doi: 10.1001/jama.2020.12603 PMC7349096 32644129

[11] Nath A. Long-haul COVID. Neurology (2020) 95(13):559–60. doi: 10.1212/WNL.0000000000010640 32788251

[12] Ferrari R Russell AS. A questionnaire using the modified 2010 american college of rheumatology criteria for fibromyalgia: specificity and sensitivity in clinical practice. J Rheumatol (2013) 40(9):1590–5. doi: 10.3899/jrheum.130367 23818707

[13] Komaroff AL Lipkin WI. Insights from myalgic encephalomyelitis/chronic fatigue syndrome may help unravel the pathogenesis of postacute COVID-19 syndrome. Trends Mol Med (2021) 27(9):895–906. doi: 10.1016/j.molmed.2021.06.002 34175230 PMC8180841

[14] Mueller C Lin JC Sheriff S Maudsley AA Younger JW. Evidence of widespread metabolite abnormalities in Myalgic encephalomyelitis/chronic fatigue syndrome: assessment with whole-brain magnetic resonance spectroscopy. Brain Imaging Behav (2020) 14(2):562–72. doi: 10.1007/s11682-018-0029-4 PMC6612467 30617782

[15] Douaud G Lee S Alfaro-Almagro F Arthofer C Wang CY McCarthy P. SARS-CoV-2 is associated with changes in brain structure in UK Biobank. Nature (2022) 604(7907):697–+. doi: 10.1038/s41586-022-04569-5 PMC9046077 35255491

[16] Pan RG Zhang QR Anthony SM Zhou Y Zou XF Cassell M. Oligodendrocytes that survive acute coronavirus infection induce prolonged inflammatory responses in the CNS. Proc Natl Acad Sci United States America. (2020) 117(27):15902–10. doi: 10.1073/pnas.2003432117 PMC7355048 32571951

[17] Ledford H. Do vaccines protect against long COVID? What the data say. Nature (2021) 559(7886):546–8. doi: 10.1038/d41586-021-03495-2 34815580

[18] Meinhardt J Radke J Dittmayer C Franz J Thomas C Mothes R. Olfactory transmucosal SARS-CoV-2 invasion as a port of central nervous system entry in individuals with COVID-19. Nat Neurosci (2021) 24(2):168–75. doi: 10.1038/s41593-020-00758-5 33257876

[19] Daniels BP Holman DW Cruz-Orengo L Jujjavarapu H Durrant DM Klein RS. Viral pathogen-associated molecular patterns regulate blood-brain barrier integrity *via* competing innate cytokine signals. mBio (2014) 5(5):e01476–14. doi: 10.1128/mBio.01476-14 PMC4173776 25161189

[20] Boldrini M Canoll PD Klein RS. How COVID-19 affects the brain. JAMA Psychiat. (2021) 78(6):682–3. doi: 10.1001/jamapsychiatry.2021.0500 PMC9894299 33769431

[21] Nalbandian A Sehgal K Gupta A Madhavan MV McGroder C Stevens JS. Post-acute COVID-19 syndrome. Nat Med (2021) 27(4):601–15. doi: 10.1038/s41591-021-01283-z PMC8893149 33753937

[22] Iosef C Knauer MJ Nicholson M Van Nynatten LR Cepinskas G Draghici S. Plasma proteome of Long-COVID patients indicates HIF-mediated vasculo-proliferative disease with impact on brain and heart function. J Trans Med (2023) 21(1):377. doi: 10.1186/s12967-023-04149-9 PMC10257382 37301958

[23] Carvalho T Krammer F Iwasaki A. The first 12 months of COVID-19: a timeline of immunological insights. Nat Rev Immunol (2021) 21(4):245–56. doi: 10.1038/s41577-021-00522-1 PMC7958099 33723416

[24] Poon DC Ho YS Chiu K Wong HL Chang RC. Sickness: From the focus on cytokines, prostaglandins, and complement factors to the perspectives of neurons. Neurosci Biobehav Rev (2015) 57:30–45. doi: 10.1016/j.neubiorev.2015.07.015 26363665

[25] Dantzer R Heijnen CJ Kavelaars A Laye S Capuron L. The neuroimmune basis of fatigue. Trends Neurosci (2014) 37(1):39–46. doi: 10.1016/j.tins.2013.10.003 24239063 PMC3889707

[26] Kennedy G Spence VA McLaren M Hill A Underwood C Belch JJ. Oxidative stress levels are raised in chronic fatigue syndrome and are associated with clinical symptoms. Free Radic Biol Med (2005) 39(5):584–9. doi: 10.1016/j.freeradbiomed.2005.04.020 16085177

[27] Mandarano AH Maya J Giloteaux L Peterson DL Maynard M Gottschalk CG. Myalgic encephalomyelitis/chronic fatigue syndrome patients exhibit altered T cell metabolism and cytokine associations. J Clin Invest (2020) 130(3):1491–505. doi: 10.1172/JCI132185 PMC7269566 31830003

[28] Naviaux RK Naviaux JC Li K Bright AT Alaynick WA Wang L. Metabolic features of chronic fatigue syndrome. Proc Natl Acad Sci United States America. (2016) 113(37):E5472–80. doi: 10.1073/pnas.1607571113 PMC5027464 27573827

[29] Vermeulen RCW Kurk RM Visser FC Sluiter W Scholte HR. Patients with chronic fatigue syndrome performed worse than controls in a controlled repeated exercise study despite a normal oxidative phosphorylation capacity. J Trans Med (2010) 8:93. doi: 10.1186/1479-5876-8-93 PMC2964609 20937116

[30] Jason LA Sunnquist M Brown A Evans M Vernon SD Furst J. Examining case definition criteria for chronic fatigue syndrome and myalgic encephalomyelitis. Fatigue (2014) 2(1):40–56. doi: 10.1080/21641846.2013.862993 24511456 PMC3912876

[31] Lim EJ Ahn YC Jang ES Lee SW Lee SH Son CG. Systematic review and meta-analysis of the prevalence of chronic fatigue syndrome/myalgic encephalomyelitis (CFS/ME). J Trans Med (2020) 18(1):100. doi: 10.1186/s12967-020-02269-0 PMC7038594 32093722

[32] Bedree H Sunnquist M Jason LA. The dePaul symptom questionnaire-2: A validation study. Fatigue (2019) 7(3):166–79. doi: 10.1080/21641846.2019.1653471 PMC7367506 32685281

[33] Gandasegui IM Laka LA Gargiulo PA Gomez-Esteban JC Sanchez JVL. Myalgic encephalomyelitis/chronic fatigue syndrome: A neurological entity? Medicina-Lithuania (2021) 57(10):1030. doi: 10.3390/medicina57101030 PMC8540700 34684066

[34] Maes M Twisk FNM. Why myalgic encephalomyelitis/chronic fatigue syndrome (ME/CFS) may kill you: disorders in the inflammatory and oxidative and nitrosative stress (IO&NS) pathways may explain cardiovascular disorders in ME/CFS. Neuroendocrinol Lett (2009) 30(6):677–93. 20038921

[35] Fraser DD Slessarev M Martin CM Daley M Patel MA Miller MR. Metabolomics profiling of critically ill coronavirus disease 2019 patients: identification of diagnostic and prognostic biomarkers. Crit Care Explor (2020) 2(10):e0272. doi: 10.1097/CCE.0000000000000272 33134953 PMC7587450

[36] Meng X Dunsmore G Koleva P Elloumi Y Wu RY Sutton RT. The profile of human milk metabolome, cytokines, and antibodies in inflammatory bowel diseases versus healthy mothers, and potential impact on the newborn. J Crohns Colitis. (2019) 13(4):431–41. doi: 10.1093/ecco-jcc/jjy186 PMC6441305 30418545

[37] Pang ZQ Chong J Zhou GY Morais DAD Chang L Barrette M. MetaboAnalyst 5.0: narrowing the gap between raw spectra and functional insights. Nucleic Acids Res (2021) 49(W1):W388–W96. doi: 10.1093/nar/gkab382 PMC8265181 34019663

[38] Xu J Zhou M Luo P Yin Z Wang S Liao T. Plasma metabolomic profiling of patients recovered from coronavirus disease 2019 (COVID-19) with pulmonary sequelae 3 months after discharge. Clin Infect Dis (2021) 73(12):2228–39. doi: 10.1093/cid/ciab147 PMC7929060 33596592

[39] Elkattawy S Alyacoub R Ayad S Pandya M Eckman A. A novel case of hypoparathyroidism secondary to SARS-coV-2 infection. Cureus (2020) 12(8):e10097. doi: 10.7759/cureus.10097 33005518 PMC7522187

[40] Liu J Han P Wu J Gong J Tian D. Prevalence and predictive value of hypocalcemia in severe COVID-19 patients. J Infect Public Health (2020) 13(9):1224–8. doi: 10.1016/j.jiph.2020.05.029 PMC7306733 32622796

[41] da Silva GB Manica D da Silva AP Kosvoski GC Hanauer M Assmann CE. High levels of extracellular ATP lead to different inflammatory responses in COVID-19 patients according to the severity. J Mol Med (Berl). (2022) 100(4):645–63. doi: 10.1007/s00109-022-02185-4 PMC8898096 35249135

[42] Shen B Yi X Sun Y Bi X Du J Zhang C. Proteomic and metabolomic characterization of COVID-19 patient sera. Cell (2020) 182(1):59–72 e15. doi: 10.1016/j.cell.2020.05.032 32492406 PMC7254001

[43] Thomas T Stefanoni D Reisz JA Nemkov T Bertolone L Francis RO. COVID-19 infection alters kynurenine and fatty acid metabolism, correlating with IL-6 levels and renal status. JCI Insight (2020) 5(14):e140327. doi: 10.1172/jci.insight.140327 32559180 PMC7453907

[44] Armstrong CW McGregor NR Sheedy JR Buttfield I Butt HL Gooley PR. NMR metabolic profiling of serum identifies amino acid disturbances in chronic fatigue syndrome. Clin Chim Acta (2012) 413(19-20):1525–31. doi: 10.1016/j.cca.2012.06.022 22728138

[45] Nagy G Barcza M Gonchoroff N Phillips PE Perl A. Nitric oxide-dependent mitochondrial biogenesis generates Ca2+ signaling profile of lupus T cells. J Immunol (2004) 173(6):3676–83. doi: 10.4049/jimmunol.173.6.3676 PMC4034140 15356113

[46] Yoshida K Furuya S Osuka S Mitoma J Shinoda Y Watanabe M. Targeted disruption of the mouse 3-phosphoglycerate dehydrogenase gene causes severe neurodevelopmental defects and results in embryonic lethality. J Biol Chem (2004) 279(5):3573–7. doi: 10.1074/jbc.C300507200 14645240

[47] Tabatabaie L Klomp LW Rubio-Gozalbo ME Spaapen LJ Haagen AA Dorland L. Expanding the clinical spectrum of 3-phosphoglycerate dehydrogenase deficiency. J Inherit Metab Dis (2011) 34(1):181–4. doi: 10.1007/s10545-010-9249-5 PMC3026672 21113737

[48] Murtas G Marcone GL Sacchi S Pollegioni L. L-serine synthesis *via* the phosphorylated pathway in humans. Cell Mol Life Sci CMLS. (2020) 77(24):5131–48. doi: 10.1007/s00018-020-03574-z PMC11105101 32594192

[49] de Koning TJ. Amino acid synthesis deficiencies. J Inherit Metab Dis (2017) 40(4):609–20. doi: 10.1007/s10545-017-0063-1 PMC5500668 28653176

[50] MacKay MB Kravtsenyuk M Thomas R Mitchell ND Dursun SM. Baker GB. D-Serine: Potential Ther Agent and/or biomark Schizophr Depression? Front Psychiatry (2019) 10:25. doi: 10.3389/fpsyt.2019.00025 PMC6372501 30787885

[51] Huang CC Wei IH Huang CL Chen KT Tsai MH Tsai P. Inhibition of glycine transporter-I as a novel mechanism for the treatment of depression. Biol Psychiat. (2013) 74(10):734–41. doi: 10.1016/j.biopsych.2013.02.020 23562005

[52] Kumar V Ahmad MA Najmi AK Akhtar M. Effect of sarcosine (a glycine transport 1 inhibitor) and risperidone (an atypical antipsychotic drug) on MK-801 induced learning and memory deficits in rats. Drug Res (Stuttg). (2016) 66(1):11–7. doi: 10.1055/s-0035-1545299 25710578

[53] Tsai CH Huang HC Liu BL Li CI Lu MK Chen X. Activation of N-methyl-D-aspartate receptor glycine site temporally ameliorates neuropsychiatric symptoms of Parkinson's disease with dementia. Psychiatry Clin Neurosci (2014) 68(9):692–700. doi: 10.1111/pcn.12175 24612097

[54] Chen KT Wu CH Tsai MH Wu YC Jou MJ Huang CC. Antidepressant-like effects of long-term sarcosine treatment in rats with or without chronic unpredictable stress. Behav Brain Res (2017) 316:1–10. doi: 10.1016/j.bbr.2016.06.004 27555541

[55] Walters RO Arias E Diaz A Burgos ES Guan FX Tiano S. Sarcosine is uniquely modulated by aging and dietary restriction in rodents and humans. Cell Rep (2018) 25(3):663–+. doi: 10.1016/j.celrep.2018.09.065 PMC6280974 30332646

[56] Lindseth G Helland B Caspers J. The effects of dietary tryptophan on affective disorders. Arch Psychiat Nurs. (2015) 29(2):102–7. doi: 10.1016/j.apnu.2014.11.008 PMC4393508 25858202

[57] Reizine F LesouHaitier M Gregoire M Pinceaux K Gacouin A Maamar A. SARS-coV-2-induced ARDS associates with MDSC expansion, lymphocyte dysfunction, and arginine shortage. J Clin Immunol (2021) 41(3):515–25. doi: 10.1007/s10875-020-00920-5 PMC7775842 33387156

[58] Shahbaz S Xu L Osman M Sligl W Shields J Joyce M. Erythroid precursors and progenitors suppress adaptive immunity and get invaded by SARS-CoV-2. Stem Cell Rep (2021) 16(5):1165–81. doi: 10.1016/j.stemcr.2021.04.001 PMC8111797 33979601

[59] Saito S Shahbaz S Sligl W Osman M Tyrrell DL Elahi S. Differential impact of SARS-coV-2 isolates, namely, the wuhan strain, delta, and omicron variants on erythropoiesis. Microbiol Spectr 2022 10(4):e0173022. doi: 10.1128/spectrum.01730-22 35943266 PMC9430111

[60] Fiorentino G Coppola A Izzo R Annunziata A Bernardo M Lombardi A. Effects of adding L-arginine orally to standard therapy in patients with COVID-19: A randomized, double-blind, placebo-controlled, parallel-group trial. Results first interim analysis. Eclinicalmedicine. (2021) 40:101125. doi: 10.1016/j.eclinm.2021.101125 34522871 PMC8428476

[61] Errico F Nuzzo T Carella M Bertolino A Usiello A. The emerging role of altered d-aspartate metabolism in schizophrenia: new insights from preclinical models and human studies. Front Psychiatry (2018) 9:559. doi: 10.3389/fpsyt.2018.00559 30459655 PMC6232865

[62] Sacchi S Novellis V Paolone G Nuzzo T Iannotta M Belardo C. Olanzapine, but not clozapine, increases glutamate release in the prefrontal cortex of freely moving mice by inhibiting D-aspartate oxidase activity. Sci Rep (2017) 7:46288. doi: 10.1038/srep46288 28393897 PMC5385520

[63] Burtscher M Brunner F Faulhaber M Hotter B Likar R. The prolonged intake of L-arginine-L-aspartate reduces blood lactate accumulation and oxygen consumption during submaximal exercise. J Sports Sci Med (2005) 4(3):314–22. PMC3887335 24453536

[64] Wang Z Ying Z Bosy-Westphal A Zhang J Schautz B Later W. Specific metabolic rates of major organs and tissues across adulthood: evaluation by mechanistic model of resting energy expenditure. Am J Clin Nutr (2010) 92(6):1369–77. doi: 10.3945/ajcn.2010.29885 PMC2980962 20962155

[65] Brealey D Brand M Hargreaves I Heales S Land J Smolenski R. Association between mitochondrial dysfunction and severity and outcome of septic shock. Lancet (2002) 360(9328):219–23. doi: 10.1016/S0140-6736(02)09459-X 12133657

[66] Lee I Huttemann M. Energy crisis: the role of oxidative phosphorylation in acute inflammation and sepsis. Biochim Biophys Acta (2014) 1842(9):1579–86. doi: 10.1016/j.bbadis.2014.05.031 PMC4147665 24905734

[67] Myhill S Booth NE McLaren-Howard J. Targeting mitochondrial dysfunction in the treatment of Myalgic Encephalomyelitis/Chronic Fatigue Syndrome (ME/CFS) - a clinical audit. Int J Clin Exp Med (2013) 6(1):1–15. 23236553 PMC3515971

[68] Castro-Marrero J Cordero MD Saez-Francas N Jimenez-Gutierrez C Aguilar-Montilla FJ Aliste L. Could mitochondrial dysfunction be a differentiating marker between chronic fatigue syndrome and fibromyalgia? Antioxid Redox Sign (2013) 19(15):1855–60. doi: 10.1089/ars.2013.5346 23600892

[69] Nakano M Imamura H Sasaoka N Yamamoto M Uemura N Shudo T. ATP maintenance *via* two types of ATP regulators mitigates pathological phenotypes in mouse models of parkinson's disease. Ebiomedicine (2017) 22:225–41. doi: 10.1016/j.ebiom.2017.07.024 PMC5552266 28780078

[70] Duan Z Lu J. Involvement of aryl hydrocarbon receptor in L-kynurenine-mediated parathyroid hormone-related peptide expression. Horm Cancer (2019) 10(2-3):89–96. doi: 10.1007/s12672-019-0357-x 30689168 PMC10355699

[71] Ha S Jin B Clemmensen B Park P Mahboob S Gladwill V. Serotonin is elevated in COVID-19-associated diarrhoea. Gut (2021) 70(10):2015–+. doi: 10.1136/gutjnl-2020-323542 PMC9208360 33402416

[72] Fonseca W Malinczak CA Schuler CF Best SKK Rasky AJ Morris SB. Uric acid pathway activation during respiratory virus infection promotes Th2 immune response *via* innate cytokine production and ILC2 accumulation. Mucosal Immunol (2020) 13(4):691–701. doi: 10.1038/s41385-020-0264-z 32047272 PMC7316593

[73] Kovarik JJ Bileck A Hagn G Meier-Menches SM Frey T Kaempf A. A multi-omics based anti-inflammatory immune signature characterizes long COVID-19 syndrome. iScience (2023) 26(1):105717. doi: 10.1016/j.isci.2022.105717 36507225 PMC9719844

[74] Omori K Katakami N Yamamoto Y Ninomiya H Takahara M Matsuoka TA. Identification of metabolites associated with onset of CAD in diabetic patients using CE-MS analysis: A pilot study. J Atheroscler Thromb (2019) 26(3):233–45. doi: 10.5551/jat.42945 PMC6402886 30068816

[75] da Silva R de Sarges KML Cantanhede MHD da Costa FP Dos Santos EF Rodrigues FBB. Thrombophilia and immune-related genetic markers in long COVID. Viruses (2023) 15(4):885. doi: 10.3390/v15040885 37112866 PMC10143911

[76] Labarrere CA Kassab GS. Glutathione: A Samsonian life-sustaining small molecule that protects against oxidative stress, ageing and damaging inflammation. Front Nutr (2022) 9. doi: 10.3389/fnut.2022.1007816 PMC9664149 36386929

[77] Rojas M Rodriguez Y Acosta-Ampudia Y Monsalve DM Zhu CS Li QZ. Autoimmun is hallmark post-COVID syndrome. J Trans Med (2022) 20(1):129. doi: 10.1186/s12967-022-03328-4 PMC8924736 35296346

[78] Su Y Yuan D Chen DG Ng RH Wang K Choi J. Multiple early factors anticipate post-acute COVID-19 sequelae. Cell (2022) 185(5):881–95 e20. doi: 10.1016/j.cell.2022.01.014 35216672 PMC8786632

[79] Wang K Khoramjoo M Srinivasan K Gordon PMK Mandal R Jackson D. Sequential multi-omics analysis identifies clinical phenotypes and predictive biomarkers for long COVID. Cell Rep Med (2023) 4(11):101254. doi: 10.1016/j.xcrm.2023.101254 37890487 PMC10694626

[80] Shahbaz S Bozorgmehr N Lu J Osman M Sligl W Tyrrell DL. Analysis of SARS-CoV-2 isolates, namely the Wuhan strain, Delta variant, and Omicron variant, identifies differential immune profiles. Microbiol Spectr. (2023) 11(5):e0125623. doi: 10.1128/spectrum.01256-23 37676005 PMC10581158

[81] Guo K Li L. Differential 12C-/13C-isotope dansylation labeling and fast liquid chromatography/mass spectrometry for absolute and relative quantification of the metabolome. Anal Chem (2009) 81(10):3919–32. doi: 10.1021/ac900166a 19309105

[82] Bozorgmehr N Mashhouri S Perez Rosero E Xu L Shahbaz S Sligl W. Galectin-9, a player in cytokine release syndrome and a surrogate diagnostic biomarker in SARS-coV-2 infection. mBio (2021) 12(3):e00384-21. doi: 10.1128/mBio.00384-21 33947753 PMC8262904

[83] Bozorgmehr N Okoye I Oyegbami O Xu L Fontaine A Cox-Kennett N. Expanded antigen-experienced CD160(+)CD8(+)effector T cells exhibit impaired effector functions in chronic lymphocytic leukemia. J Immunother Cancer (2021) 9(4):e002189. doi: 10.1136/jitc-2020-002189 33931471 PMC8098955

[84] Bozorgmehr N Syed H Mashhouri S Walker J Elahi S. Transcriptomic profiling of peripheral blood cells in HPV-associated carcinoma patients receiving combined valproic acid and avelumab. Mol Oncol (2023). doi: 10.1002/1878-0261.13519 PMC11077001 37681284

[85] Shahbaz S Okoye I Blevins G Elahi S. Elevated ATP *via* enhanced miRNA-30b, 30c, and 30e downregulates the expression of CD73 in CD8+ T cells of HIV-infected individuals. PloS Pathog (2022) 18(3):e1010378. doi: 10.1371/journal.ppat.1010378 35325005 PMC8947394

