# Supplementary material for: Metabolomic and immune alterations in long COVID patients with chronic fatigue syndrome
Source: Front Immunol. 2024 Jan 18;15:1341843. doi: 10.3389/fimmu.2024.1341843 (PMC10830702; doi:10.3389/fimmu.2024.1341843)
Supplement: Supplementary file 1 [file DataSheet_1.pdf]

Supplementary Table 1. Demographic, clinical and sampling schedules.

Patient ID	Age	Sex	Hospitalization	PCR test date/initial sampling	Previous health issues	Time of post-COVID sampling	Months post-COVID sampling
LC1	36	F	No	11/05/2020		07/05/2021	12
LC2	63	F	Hospital ward	11/04/2020		15/04/2021	12
LC3	45	M	No	19/11/2020		22/11/2021	12
LC4	39	M	No	28/07/2020		25/07/2021	12
LC5	52	F	No	07/03/2020		10/03/2021	12
LC6	74	F	No	26/03/2020		10/04/2021	12.5
LC7	55	F	Hospital ward	07/03/2020		28/07/2021	16.5
LC8	51	F	Hospital ward	25/07/2020		28/07/2021	12
LC10	47	F	No	15/07/2020		28/07/2021	12.5
LC12	57	M	No	12/02/2020		5/02/2021	12
LC13	56	F	No	14/04/2020		9/04/2021	12
LC14	56	F	No	30/09/2020		23/09/2021	12
LC15	56	M	No	26/3/2020	Asthma	31/03/2021	12
LC16	46	F	No	12/08/2022		17/08/2021	12
LC17	61	M	No	12/08/2020		17/08/2021	12
LC18	39	F	No	3/03/2020		19/03/2021	12.5
LC19	69	F	No	14/12/2020		30/11/2021	11.5
LC20	38	F	No	24/03/2020		29/03/2021	12
LC21	63	F	No	18/11/2020		23/11/2021	12
LC22	38	F	No	30/03/2020		29/03/2021	12
LC23	38	F	ICU	27/10/2020		21/10/2021	12
LC24	82	M	Hospital ward	18/11/2020		29/11/2021	12
LC25	53	F	No	11/05/2020		17/05/2021	12
LC26	57	F	Hospital ward	13/12/2020		07/12/2021	12
LC27	26	F	No	29/12/2020		07/12/2021	11.5
LC28	47	F	No	02/03/2020		10/03/2021	12
LC29	63	F	No	30/11/2020	Inflammatory Arthritis	07/12/2021	12
LC30	43	F	No	04/04/2020		08/04/2021	12
LC31	54	F	No	11/05/2020		17/05/2021	12
LC32	54	F	No	11/04/2020		08/04/2021	12
R1	33	M	No	01/06/2020		16/06/2021	12.5
R2	30	F	No	19/11/2020	Lupus	18/11/2021	12
R3	65	F	Hospital ward	01/09/2020	Reactive Arthropathy	10/09/2021	12
R4	34	F	No	12/08/2020		12/08/2021	12
R5	56	M	No	12/10/2020		18/10/2021	12
R6	28	F	No	02/04/2020		11/04/2021	12
R7	72	M	Hospital ward	10/03/2020		8/03/2021	12

R8	28	F	No	09/04/2020		15/04/2021	12
R9	50	M	No	30/11/2020		28/11/2021	12
R10	51	F	No	24/11/2020		28/11/2021	12
R11	35	F	No	09/06/2020		16/06/2021	12
R12	52	M	No	01/05/2020		11/05/2021	12
R13	61	F	No	17/12/2020		15/12/2021	12
R14	69	F	No	21/04/2020		11/04/2021	11.5
R15	54	F	Hospital ward	12/10/2020		18/10/2021	12
HC1	40	M	NA	NA		NA	NA
HC2	31	F	NA	NA		NA	NA
HC3	42	F	NA	NA		NA	NA
HC4	56	F	NA	NA		NA	NA
HC5	54	F	NA	NA		NA	NA
HC6	26	M	NA	NA		NA	NA
HC7	57	M	NA	NA	Diabetes	NA	NA
HC8	33	F	NA	NA		NA	NA
HC9	50	F	NA	NA		NA	NA
HC10	51	M	NA	NA		NA	NA
HC11	81	F	NA	NA		NA	NA
HC12	62	F	NA	NA		NA	NA
HC13	61	M	NA	NA		NA	NA
HC14	45	F	NA	NA		NA	NA
HC15	52	F	NA	NA	Diabetes	NA	NA
A1	55	F	ICU	NA		NA	NA
A2	62	F	ICU	NA		NA	NA
A3	61	M	ICU	NA		NA	NA
A4	39	M	ICU	NA		NA	NA
A5	45	F	ICU	NA		NA	NA
A6	55	M	ICU	NA		NA	NA
A7	48	F	ICU	NA		NA	NA
A8	43	F	ICU	NA		NA	NA
A9	46	M	ICU	NA		NA	NA
A10	44	F	ICU	NA		NA	NA
A11	66	F	ICU	NA		NA	NA
A12	41	M	ICU	NA		NA	NA
A13	53	M	ICU	NA		NA	NA
A14	71	F	ICU	NA		NA	NA
A15	66	F	ICU	NA		NA	NA

Long-COVID (LC), Recovered (R), healthy control (HC) and acute COVID-19 (A).
Not performed (NP). Not applicable (NA).
