# Supplementary material for: Metabolomic and immune alterations in long COVID patients with chronic fatigue syndrome
Source: Front Immunol. 2024 Jan 18;15:1341843. doi: 10.3389/fimmu.2024.1341843 (PMC10830702; doi:10.3389/fimmu.2024.1341843)
Supplement: Supplementary Figure 1 — (A) The heatmap of the top 100 altered metabolites in acute COVID-19 patients (A) vs HC. (B) A vs R, and (C) HC vs R. [file DataSheet_3.pdf]

S Fig. 1

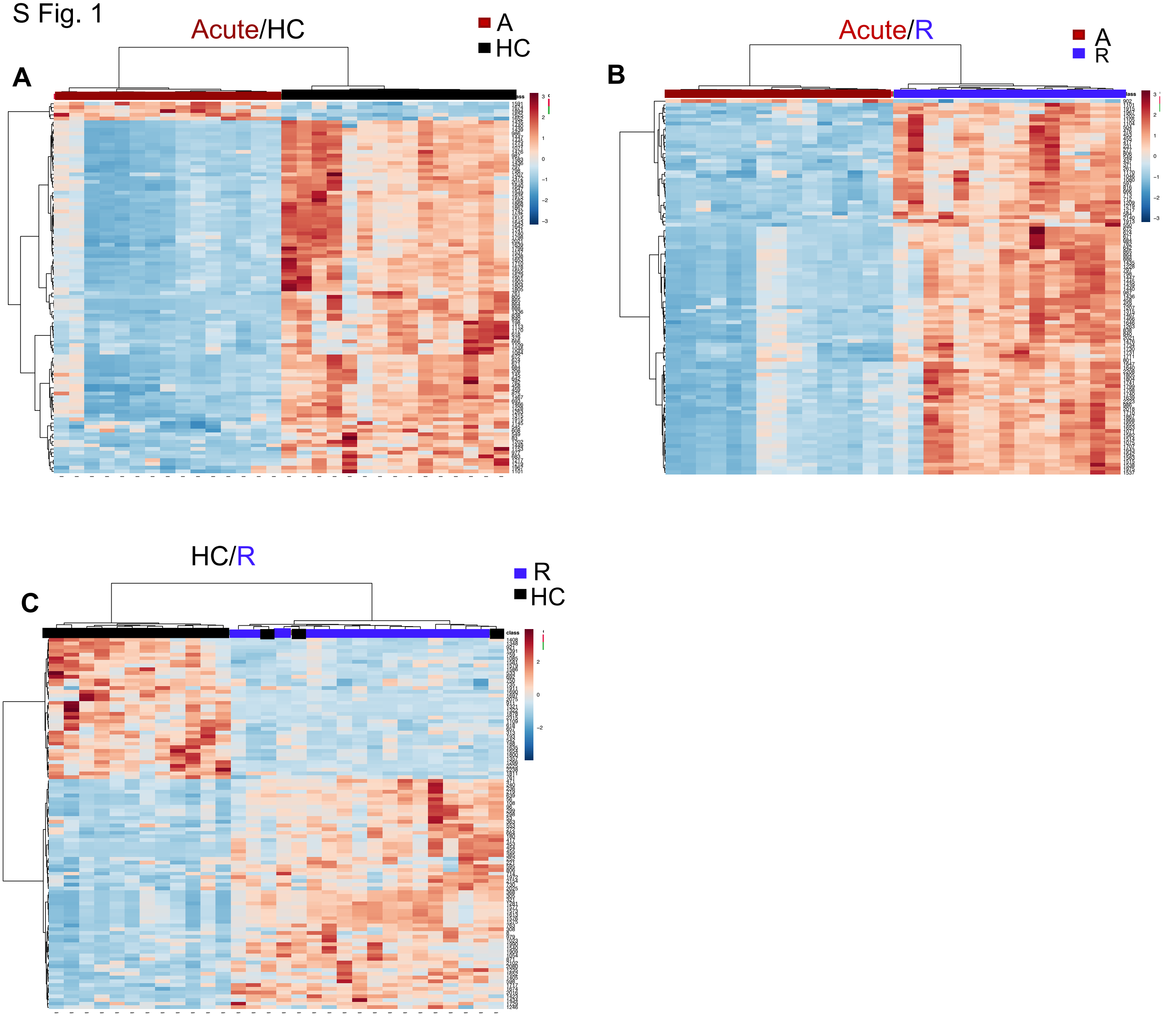

Top 100 altered metabolites in each study group

S Fig. 2

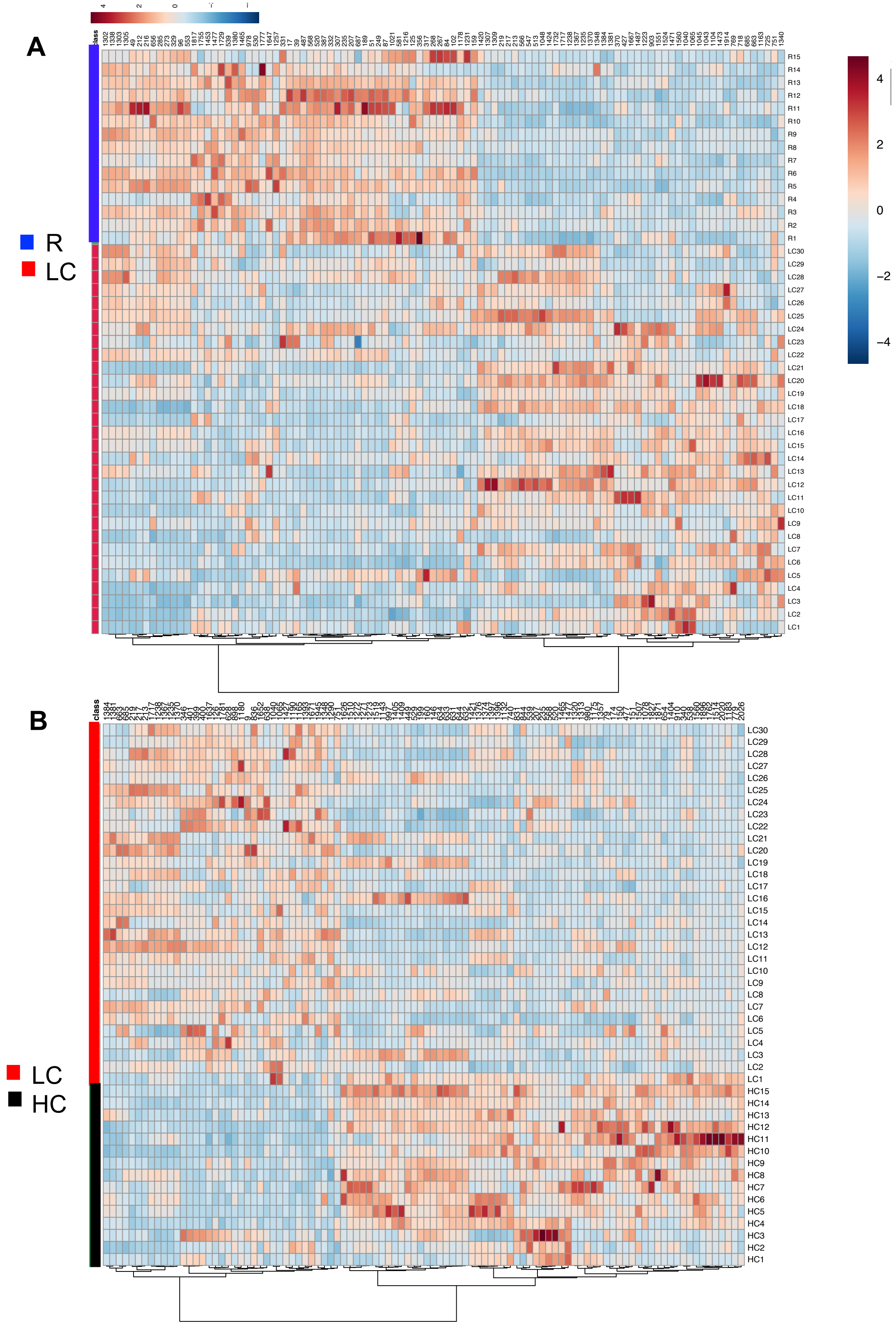

A
R
LC
class
4
2
0
−2
−4
R15
R14
R13
R12
R11
R10
R9
R8
R7
R6
R5
R4
R3
R2
R1
LC30
LC29
LC28
LC27
LC26
LC25
LC24
LC23
LC22
LC21
LC20
LC19
LC18
LC17
LC16
LC15
LC14
LC13
LC12
LC11
LC10
LC9
LC8
LC7
LC6
LC5
LC4
LC3
LC2
LC1
B
LC
HC
class
LC30
LC29
LC28
LC27
LC26
LC25
LC24
LC23
LC22
LC21
LC20
LC19
LC18
LC17
LC16
LC15
LC14
LC13
LC12
LC11
LC10
LC9
LC8
LC7
LC6
LC5
LC4
LC3
LC2
LC1
HC15
HC14
HC13
HC12
HC11
HC10
HC9
HC8
HC7
HC6
HC5
HC4
HC3
HC2
HC1

S Fig. 3

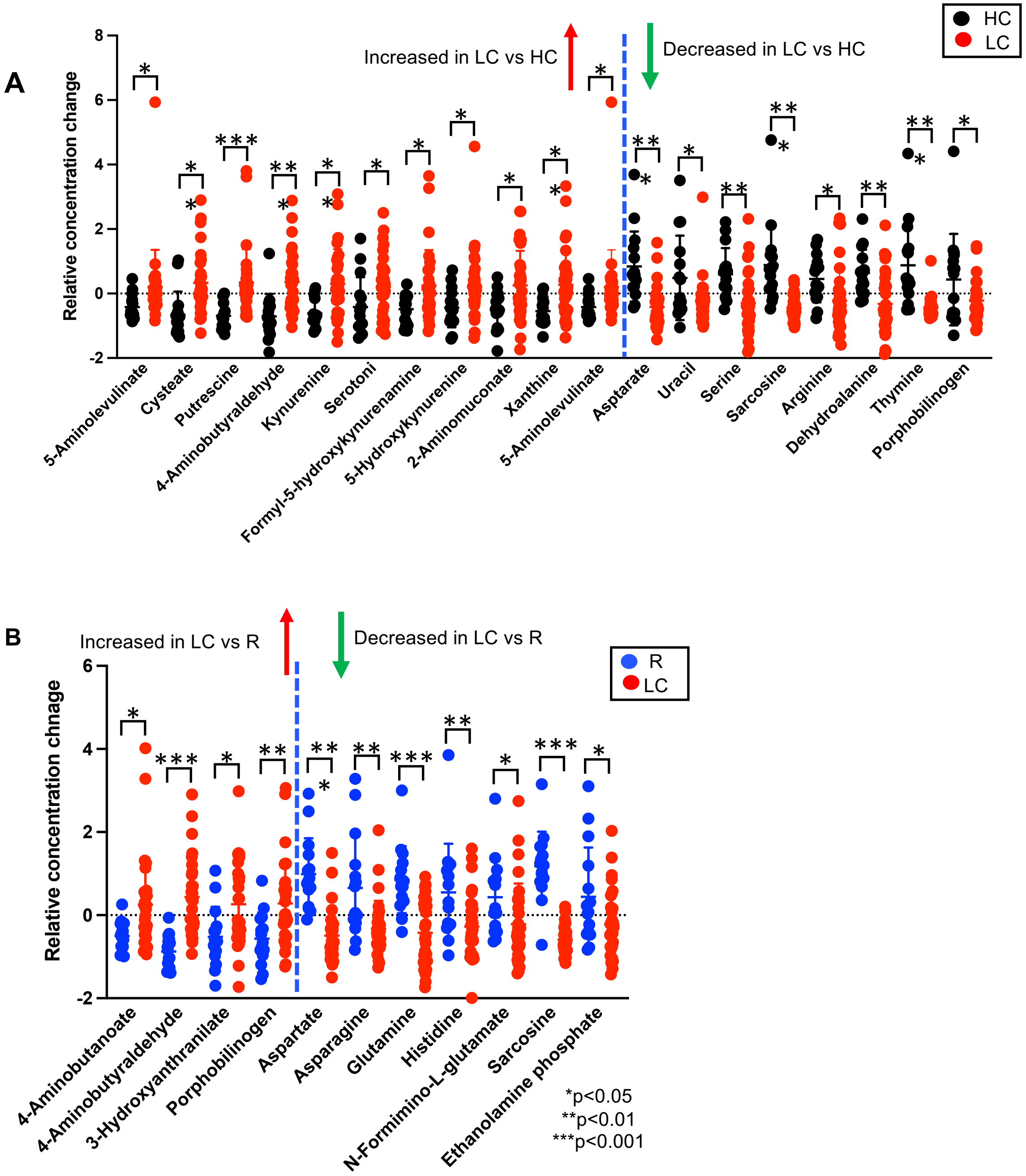
A
Increased in LC vs HC
Decreased in LC vs HC
HC
LC
Relative concentration change
5-Aminolevulinate
Cysteate
Putrescine
4-Aminobutyraldehyde
Kynurenine
Serotoni
Formyl-5-hydroxykynurenamine
5-Hydroxykynurenine
2-Aminomuconate
Xanthine
5-Aminolevulinate
Asptarate
Uracil
Serine
Sarcosine
Arginine
Dehydroalanine
Thymine
Porphobilinogen
B
Increased in LC vs R
Decreased in LC vs R
R
LC
Relative concentration chnage
4-Aminobutanoate
4-Aminobutyraldehyde
3-Hydroxyanthranilate
Porphobilinogen
Aspartate
Asparagine
Glutamine
Histidine
N-Formimino-L-glutamate
Sarcosine
Ethanolamine phosphate
*p<0.05
**p<0.01
***p<0.001

S Fig. 4

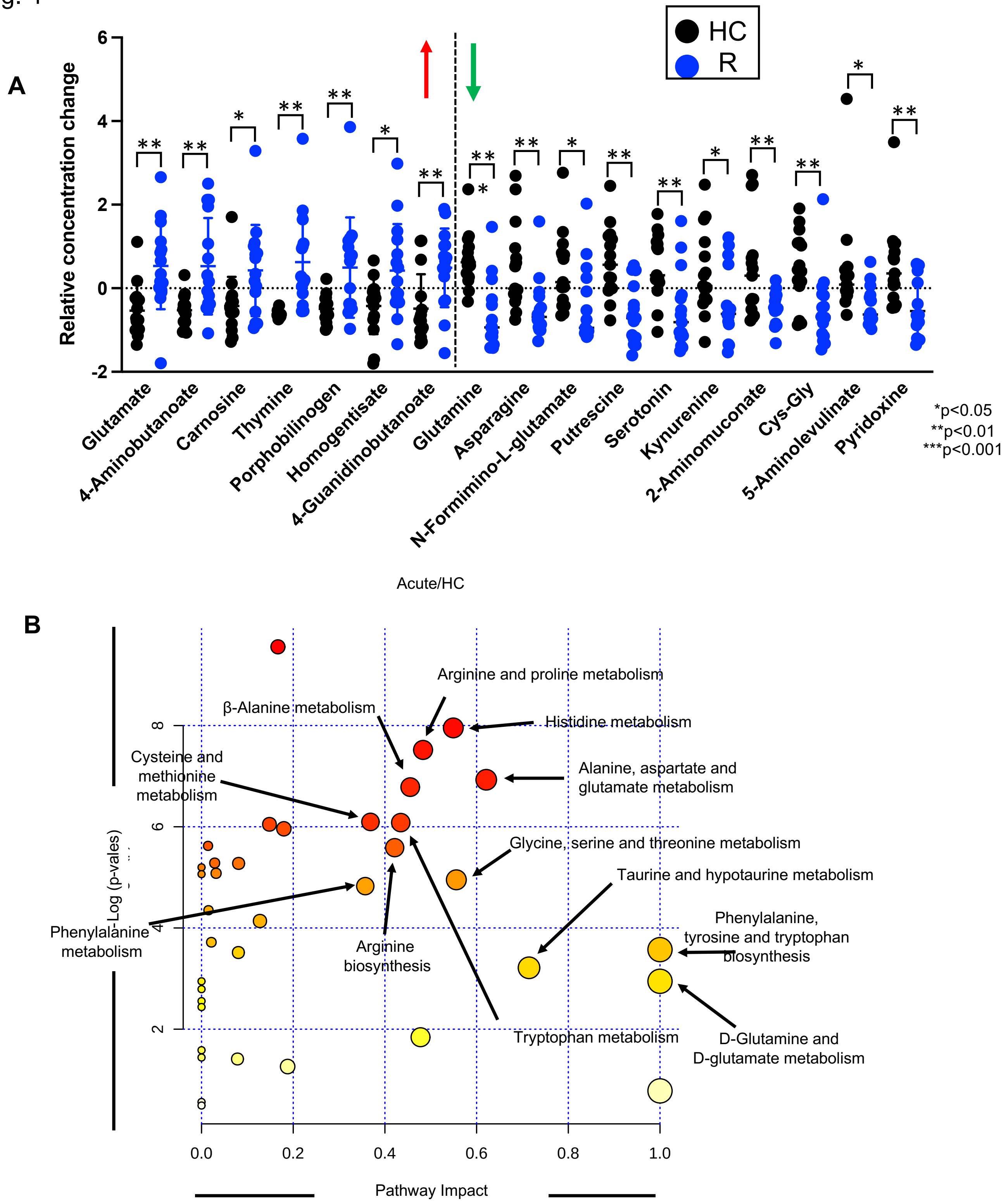
A
HC
R
Relative concentration change
6
4
2
0
-2
Glutamate
4-Aminobutanoate
Carnosine
Thymine
Porphobilinogen
Homogentisate
4-Guanidinobutanoate
Glutamine
Asparagine
N-Formimino-L-glutamate
Putrescine
Serotonin
Kynurenine
2-Aminomuconate
Cys-Gly
5-Aminolevulinate
Pyridoxine
*p<0.05
**p<0.01
***p<0.001
Acute/HC
B
-Log (p-vales)
Pathway Impact
0.0
0.2
0.4
0.6
0.8
1.0
Arginine and proline metabolism
β-Alanine metabolism
Histidine metabolism
Cysteine and methionine metabolism
Alanine, aspartate and glutamate metabolism
Glycine, serine and threonine metabolism
Taurine and hypotaurine metabolism
Phenylalanine metabolism
Arginine biosynthesis
Phenylalanine, tyrosine and tryptophan biosynthesis
Tryptophan metabolism
D-Glutamine and D-glutamate metabolism

S Fig. 5

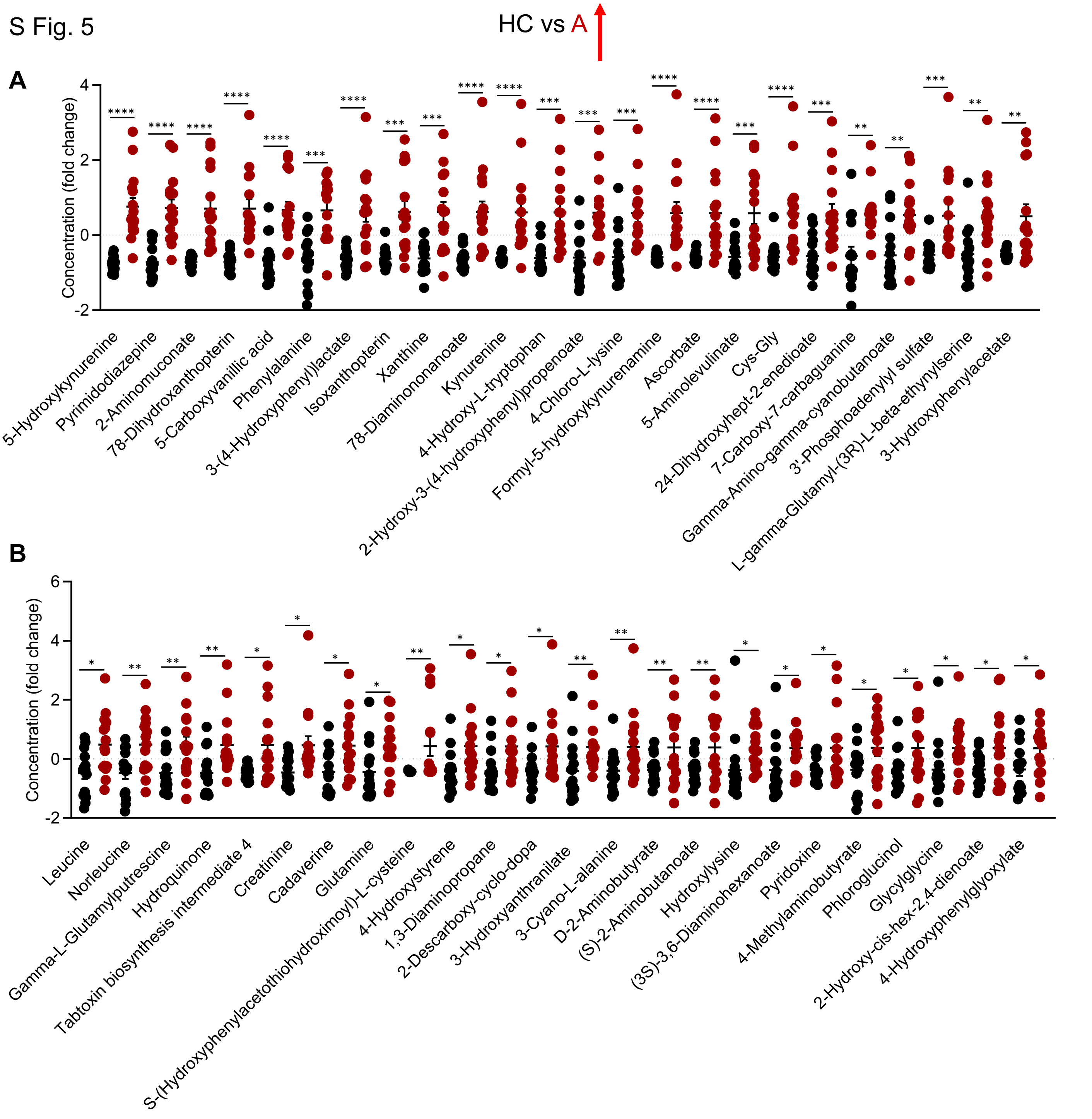
HC vs A
A
Concentration (fold change)
4
2
0
-2
5-Hydroxykynurenine
Pyrimidodiazepine
2-Aminomuconate
78-Dihydroxanthopterin
5-Carboxyvanillic acid
Phenylalanine
3-(4-Hydroxyphenyl)lactate
Isoxanthopterin
Xanthine
78-Diaminononanoate
Kynurenine
4-Hydroxy-L-tryptophan
2-Hydroxy-3-(4-hydroxyphenyl)propenoate
4-Chloro-L-lysine
Formyl-5-hydroxykynurenamine
Ascorbate
5-Aminolevulinate
Cys-Gly
24-Dihydroxyhept-2-enedioate
7-Carboxy-7-carbaguanine
Gamma-Amino-gamma-cyanobutanoate
3'-Phosphoadenylyl sulfate
L-gamma-Glutamyl-(3R)-L-beta-ethynylserine
3-Hydroxyphenylacetate
B
Concentration (fold change)
6
4
2
0
-2
Leucine
Norleucine
Gamma-L-Glutamylputrescine
Hydroquinone
Tabtoxin biosynthesis intermediate 4
Creatinine
Cadaverine
Glutamine
S-(Hydroxyphenylacetothiohydroximoyl)-L-cysteine
4-Hydroxystyrene
1,3-Diaminopropane
2-Descarboxy-cyclo-dopa
3-Hydroxyanthranilate
3-Cyano-L-alanine
D-2-Aminobutyrate
(S)-2-Aminobutanoate
Hydroxylysine
(3S)-3,6-Diaminohexanoate
Pyridoxine
4-Methylaminobutyrate
Phloroglucinol
Glycylglycine
2-Hydroxy-cis-hex-2,4-dienoate
4-Hydroxyphenylglyoxylate

S Fig. 6

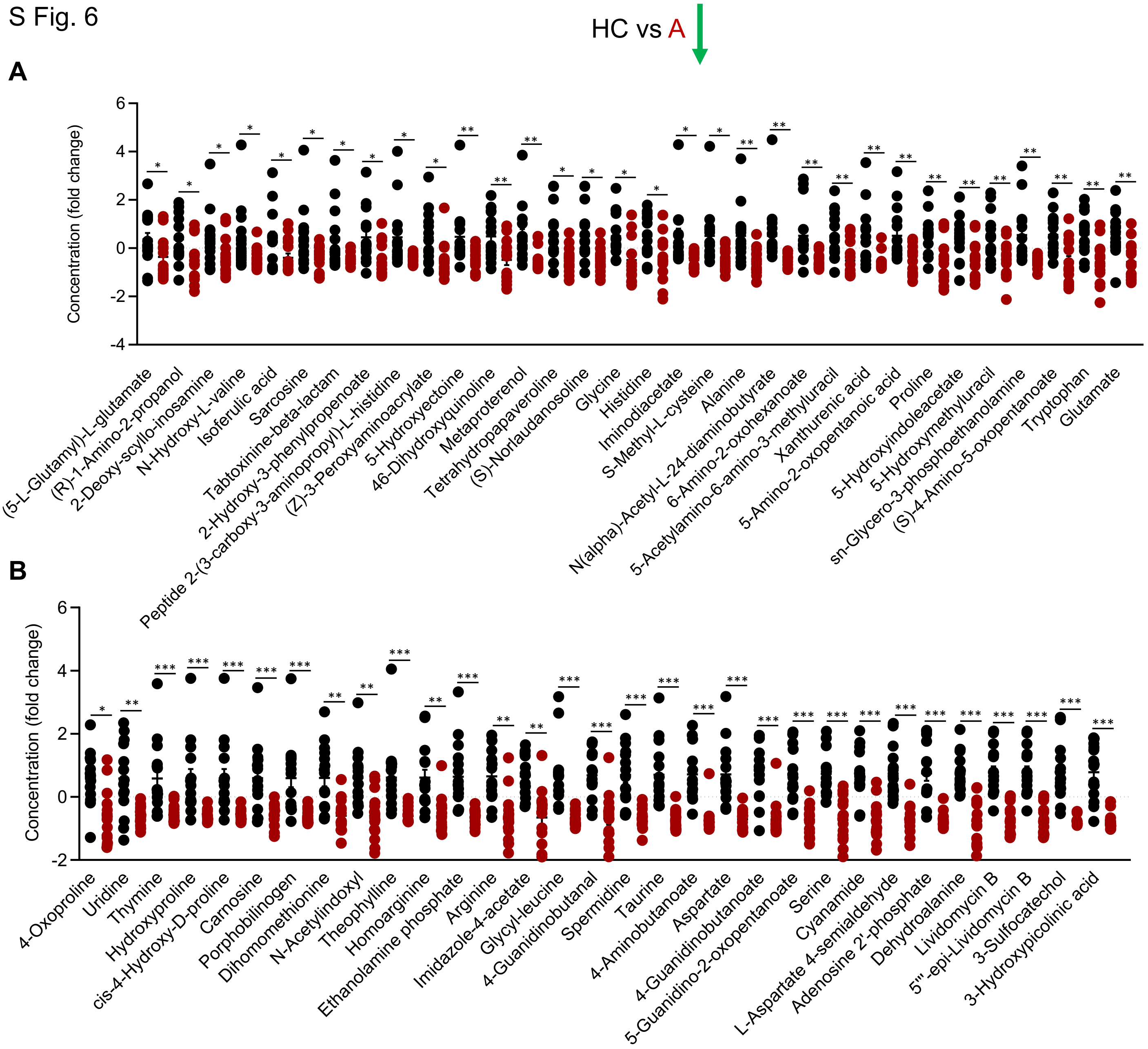
HC vs A
A
Concentration (fold change)
(5-L-Glutamyl)-L-glutamate
(R)-1-Amino-2-propanol
2-Deoxy-scyllo-inosamine
N-Hydroxy-L-valine
Isoferulic acid
Sarcosine
Tabtoxinine-beta-lactam
2-Hydroxy-3-phenylpropenoate
Peptide 2-(3-carboxy-3-aminopropyl)-L-histidine
(Z)-3-Peroxyaminoacrylate
5-Hydroxyectoine
46-Dihydroxyquinoline
Metaproterenol
Tetrahydropapaveroline
(S)-Norlaudanosoline
Glycine
Histidine
Iminodiacetate
S-Methyl-L-cysteine
Alanine
N(alpha)-Acetyl-L-24-diaminobutyrate
6-Amino-2-oxohexanoate
5-Acetylamino-6-amino-3-methyluracil
Xanthurenic acid
5-Amino-2-oxopentanoic acid
Proline
5-Hydroxyindoleacetate
5-Hydroxymethyluracil
sn-Glycero-3-phosphoethanolamine
(S)-4-Amino-5-oxopentanoate
Tryptophan
Glutamate
B
Concentration (fold change)
4-Oxoproline
Uridine
Thymine
Hydroxyproline
cis-4-Hydroxy-D-proline
Carnosine
Porphobilinogen
Dihomomethionine
N-Acetylindoxyl
Theophylline
Homoarginine
Ethanolamine phosphate
Arginine
Imidazole-4-acetate
Glycyl-leucine
4-Guanidinobutanal
Spermidine
Taurine
4-Aminobutanoate
Aspartate
4-Guanidinobutanoate
5-Guanidino-2-oxopentanoate
Serine
Cyanamide
L-Aspartate 4-semialdehyde
Adenosine 2'-phosphate
Dehydroalanine
Lividomycin B
5'''-epi-Lividomycin B
3-Sulfocatechol
3-Hydroxypicolinic acid

S Fig. 7

A vs R

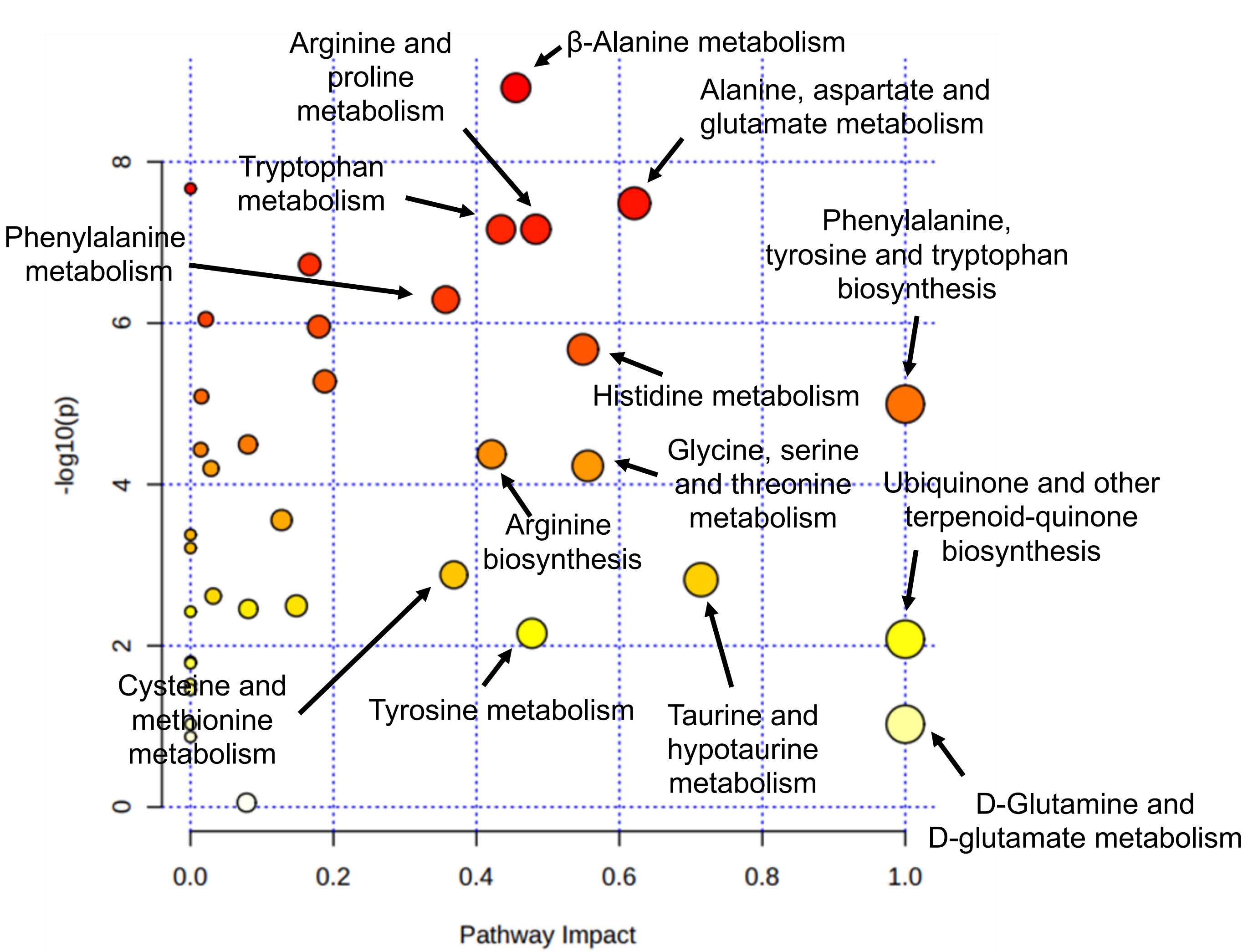
β-Alanine metabolism
Arginine and proline metabolism
Alanine, aspartate and glutamate metabolism
Tryptophan metabolism
Phenylalanine metabolism
Phenylalanine, tyrosine and tryptophan biosynthesis
Histidine metabolism
Glycine, serine and threonine metabolism
Ubiquinone and other terpenoid-quinone biosynthesis
Arginine biosynthesis
Cysteine and methionine metabolism
Tyrosine metabolism
Taurine and hypotaurine metabolism
D-Glutamine and D-glutamate metabolism
-log10(p)
0
2
4
6
8
0.0
0.2
0.4
0.6
0.8
1.0
Pathway Impact

S Fig. 8

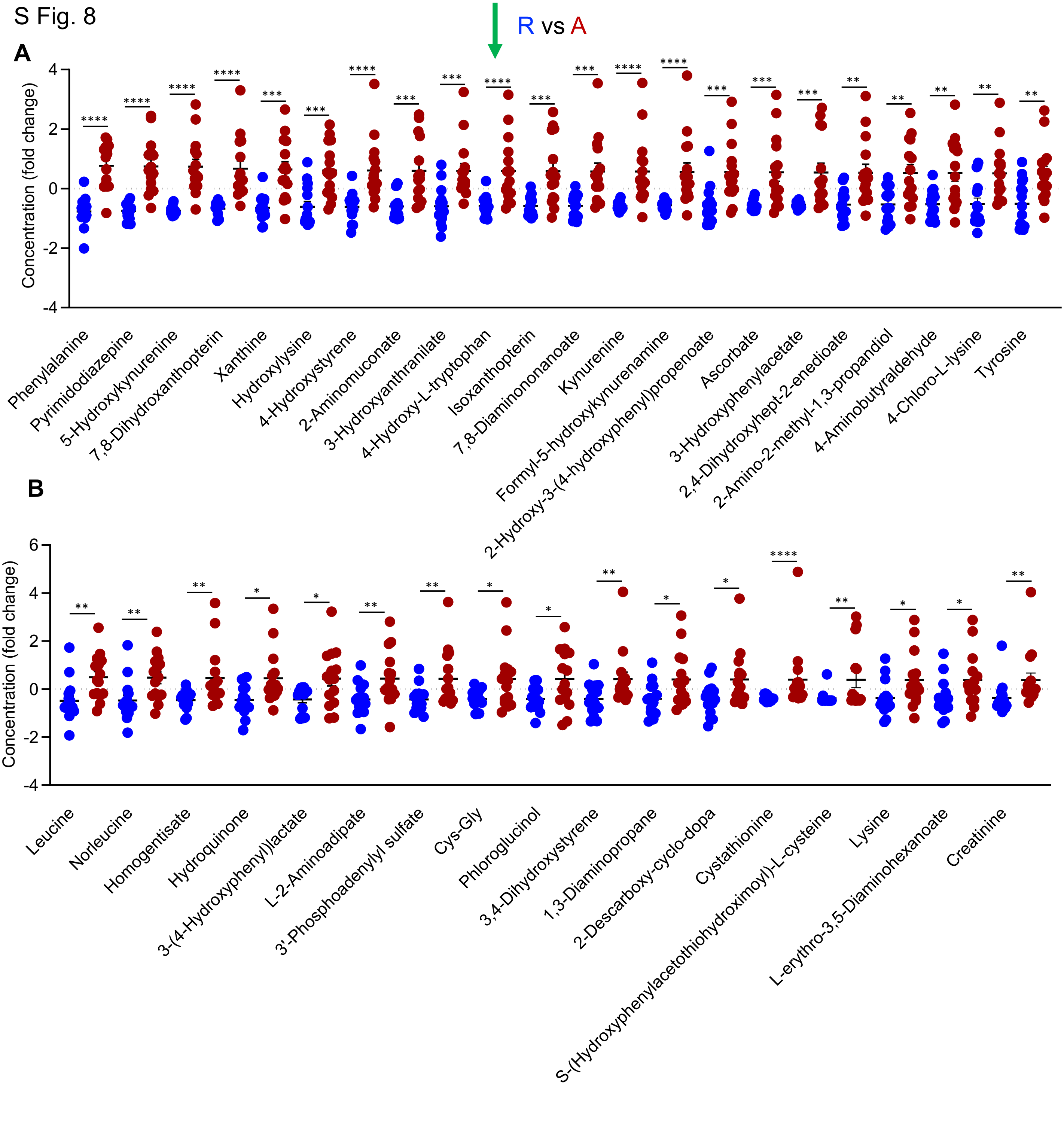
R vs A
A
Concentration (fold change)
4
2
0
-2
-4
****
****
****
****
***
***
****
***
***
****
***
***
****
****
***
***
***
**
**
**
**
**
Phenylalanine
Pyrimidodiazepine
5-Hydroxykynurenine
7,8-Dihydroxanthopterin
Xanthine
Hydroxylysine
4-Hydroxystyrene
2-Aminomuconate
3-Hydroxyanthranilate
4-Hydroxy-L-tryptophan
Isoxanthopterin
7,8-Diaminononanoate
Kynurenine
Formyl-5-hydroxykynurenamine
2-Hydroxy-3-(4-hydroxyphenyl)propenoate
Ascorbate
3-Hydroxyphenylacetate
2,4-Dihydroxyhept-2-enedioate
2-Amino-2-methyl-1,3-propandiol
4-Aminobutyraldehyde
4-Chloro-L-lysine
Tyrosine
B
Concentration (fold change)
6
4
2
0
-2
-4
**
**
**
*
*
**
**
*
*
**
*
*
****
**
*
*
**
Leucine
Norleucine
Homogentisate
Hydroquinone
3-(4-Hydroxyphenyl)lactate
L-2-Aminoadipate
3'-Phosphoadenylyl sulfate
Cys-Gly
Phloroglucinol
3,4-Dihydroxystyrene
1,3-Diaminopropane
2-Descarboxy-cyclo-dopa
Cystathionine
S-(Hydroxyphenylacetothiohydroximoyl)-L-cysteine
Lysine
L-erythro-3,5-Diaminohexanoate
Creatinine

S Fig. 9

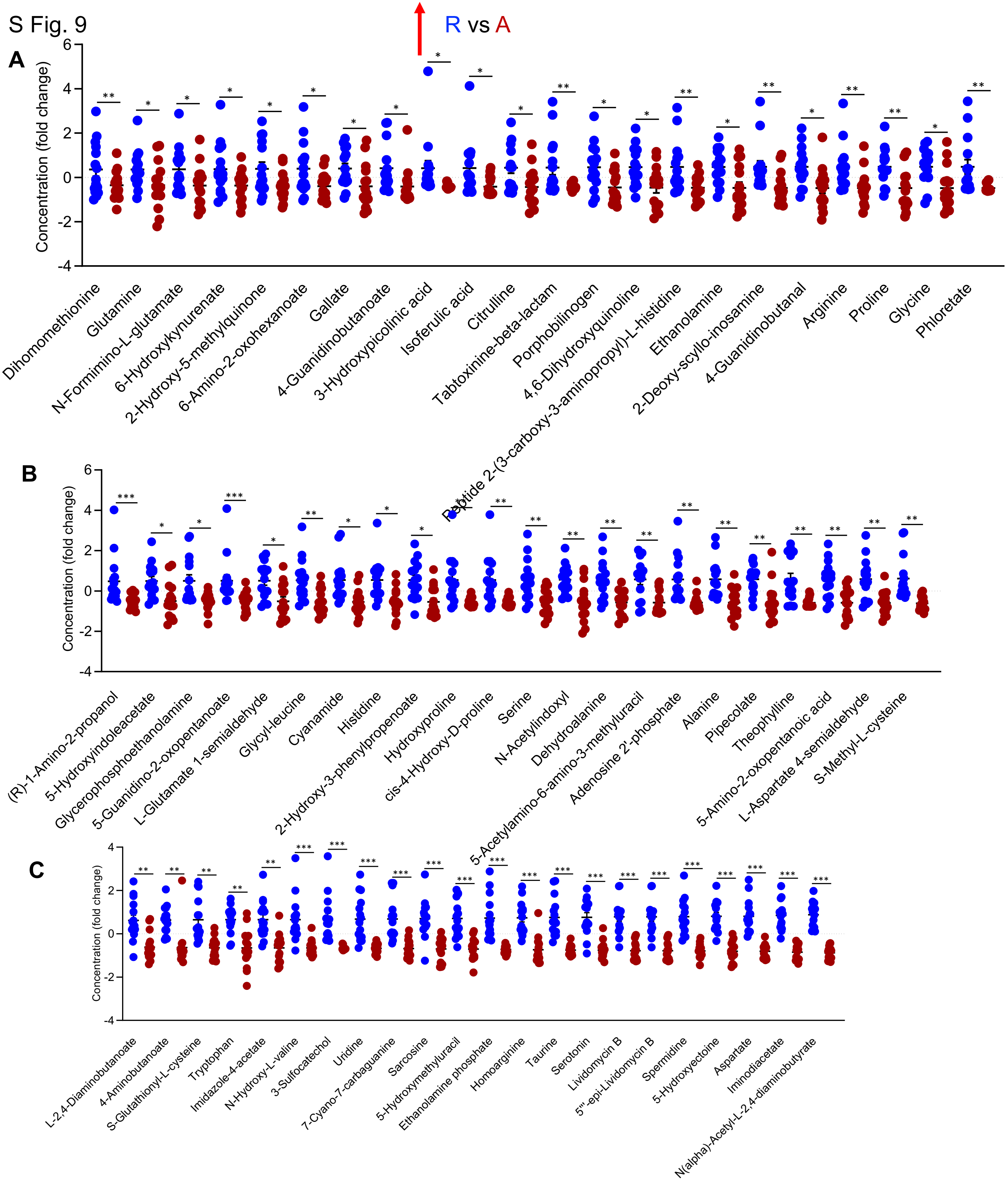

R vs A
A
Concentration (fold change)
6
4
2
0
-2
-4
*
**
***
Dihomomethionine
Glutamine
N-Formimino-L-glutamate
6-Hydroxykynurenate
2-Hydroxy-5-methylquinone
6-Amino-2-oxohexanoate
Gallate
4-Guanidinobutanoate
3-Hydroxypicolinic acid
Isoferulic acid
Citrulline
Tabtoxinine-beta-lactam
Porphobilinogen
4,6-Dihydroxyquinoline
Peptide 2-(3-carboxy-3-aminopropyl)-L-histidine
Ethanolamine
2-Deoxy-scyllo-inosamine
4-Guanidinobutanal
Arginine
Proline
Glycine
Phloretate
B
(R)-1-Amino-2-propanol
5-Hydroxyindoleacetate
Glycerophosphoethanolamine
5-Guanidino-2-oxopentanoate
L-Glutamate 1-semialdehyde
Glycyl-leucine
Cyanamide
Histidine
2-Hydroxy-3-phenylpropenoate
Hydroxyproline
cis-4-Hydroxy-D-proline
Serine
N-Acetylindoxyl
Dehydroalanine
5-Acetylamino-6-amino-3-methyluracil
Adenosine 2'-phosphate
Alanine
Pipecolate
Theophylline
5-Amino-2-oxopentanoic acid
L-Aspartate 4-semialdehyde
S-Methyl-L-cysteine
C
L-2,4-Diaminobutanoate
4-Aminobutanoate
S-Glutathionyl-L-cysteine
Tryptophan
Imidazole-4-acetate
N-Hydroxy-L-valine
3-Sulfocatechol
Uridine
7-Cyano-7-carbaguanine
Sarcosine
5-Hydroxymethyluracil
Ethanolamine phosphate
Homoarginine
Taurine
Serotonin
Lividomycin B
5'''-epi-Lividomycin B
Spermidine
5-Hydroxyectoine
Aspartate
Iminodiacetate
N(alpha)-Acetyl-L-2,4-diaminobutyrate

S Fig. 10

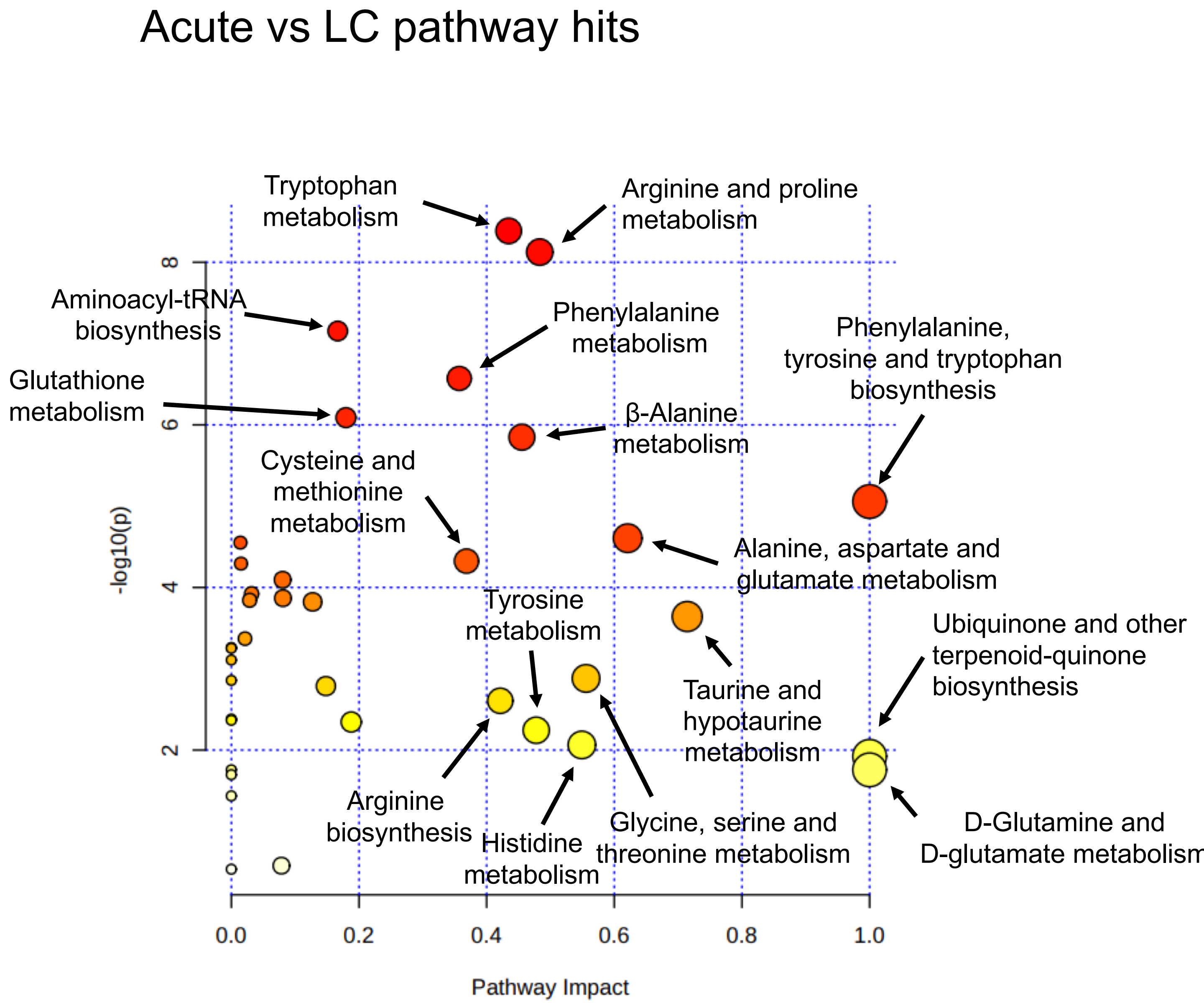

Acute vs LC pathway hits
Tryptophan metabolism
Arginine and proline metabolism
Aminoacyl-tRNA biosynthesis
Phenylalanine metabolism
Phenylalanine, tyrosine and tryptophan biosynthesis
Glutathione metabolism
β-Alanine metabolism
Cysteine and methionine metabolism
Alanine, aspartate and glutamate metabolism
Tyrosine metabolism
Taurine and hypotaurine metabolism
Ubiquinone and other terpenoid-quinone biosynthesis
Arginine biosynthesis
Histidine metabolism
Glycine, serine and threonine metabolism
D-Glutamine and D-glutamate metabolism
-log10(p)
8
6
4
2
0.0
0.2
0.4
0.6
0.8
1.0
Pathway Impact

S Fig. 11

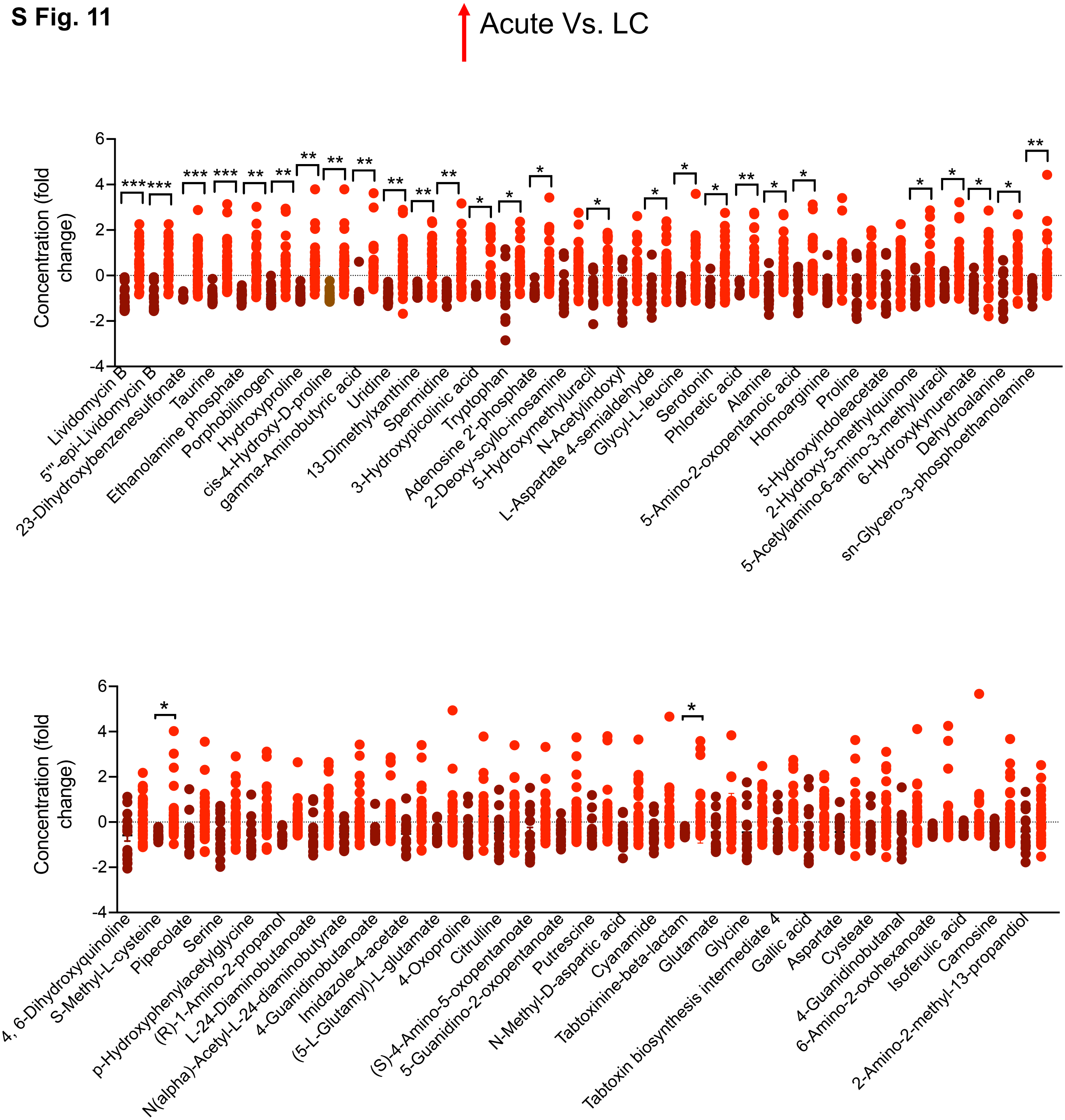
Acute Vs. LC
Concentration (fold change)
6
4
2
0
-2
-4
*** *** *** *** ** ** ** ** ** ** ** ** * * * * * * * * ** * * * * * * **
Lividomycin B
5''-epi-Lividomycin B
23-Dihydroxybenzenesulfonate
Taurine
Ethanolamine phosphate
Porphobilinogen
Hydroxyproline
cis-4-Hydroxy-D-proline
gamma-Aminobutyric acid
Uridine
13-Dimethylxanthine
Spermidine
3-Hydroxypicolinic acid
Tryptophan
Adenosine 2'-phosphate
2-Deoxy-scyllo-inosamine
5-Hydroxymethyluracil
N-Acetylindoxyl
L-Aspartate 4-semialdehyde
Glycyl-L-leucine
Serotonin
Phloretic acid
Alanine
5-Amino-2-oxopentanoic acid
Homoarginine
Proline
5-Hydroxyindoleacetate
2-Hydroxy-5-methylquinone
5-Acetylamino-6-amino-3-methyluracil
6-Hydroxykynurenate
Dehydroalanine
sn-Glycero-3-phosphoethanolamine
Concentration (fold change)
6
4
2
0
-2
-4
* *
4, 6-Dihydroxyquinoline
S-Methyl-L-cysteine
Pipecolate
Serine
p-Hydroxyphenylacetylglycine
(R)-1-Amino-2-propanol
L-24-Diaminobutanoate
N(alpha)-Acetyl-L-24-diaminobutyrate
4-Guanidinobutanoate
Imidazole-4-acetate
(5-L-Glutamyl)-L-glutamate
4-Oxoproline
Citrulline
(S)-4-Amino-5-oxopentanoate
5-Guanidino-2-oxopentanoate
Putrescine
N-Methyl-D-aspartic acid
Cyanamide
Tabtoxinine-beta-lactam
Glutamate
Glycine
Tabtoxin biosynthesis intermediate 4
Gallic acid
Aspartate
Cysteate
4-Guanidinobutanal
6-Amino-2-oxohexanoate
Isoferulic acid
Carnosine
2-Amino-2-methyl-13-propandiol

S Fig. 12

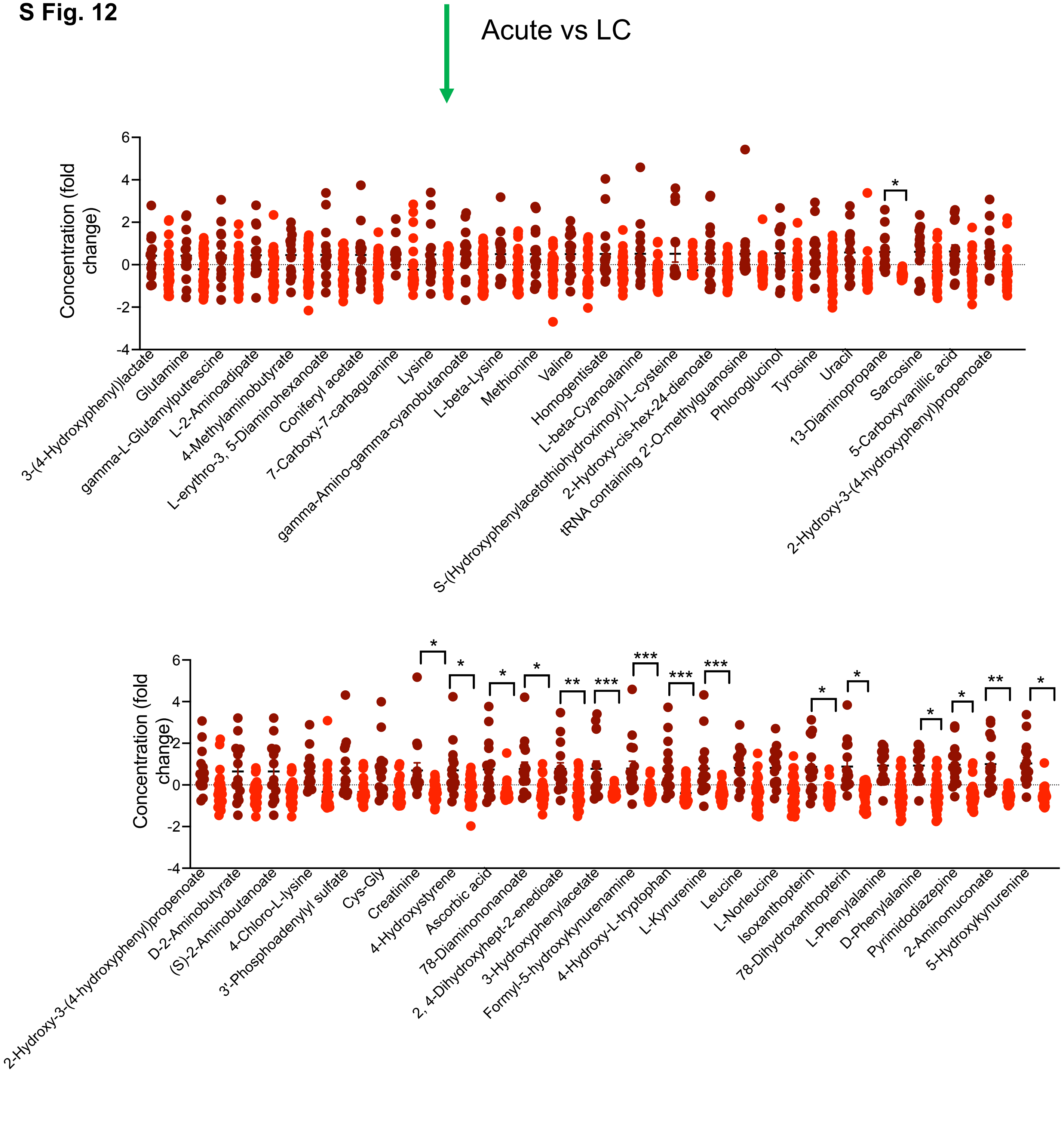
Acute vs LC
Concentration (fold change)
3-(4-Hydroxyphenyl)lactate
Glutamine
gamma-L-Glutamylputrescine
L-2-Aminoadipate
4-Methylaminobutyrate
L-erythro-3, 5-Diaminohexanoate
Coniferyl acetate
7-Carboxy-7-carbaguanine
Lysine
gamma-Amino-gamma-cyanobutanoate
L-beta-Lysine
Methionine
Valine
Homogentisate
L-beta-Cyanoalanine
S-(Hydroxyphenylacetothiohydroximoyl)-L-cysteine
2-Hydroxy-cis-hex-24-dienoate
tRNA containing 2'-O-methylguanosine
Phloroglucinol
Tyrosine
Uracil
13-Diaminopropane
Sarcosine
5-Carboxyvanillic acid
2-Hydroxy-3-(4-hydroxyphenyl)propenoate
Concentration (fold change)
2-Hydroxy-3-(4-hydroxyphenyl)propenoate
D-2-Aminobutyrate
(S)-2-Aminobutanoate
4-Chloro-L-lysine
3'-Phosphoadenylyl sulfate
Cys-Gly
Creatinine
4-Hydroxystyrene
Ascorbic acid
78-Diaminononanoate
2, 4-Dihydroxyhept-2-enedioate
3-Hydroxyphenylacetate
Formyl-5-hydroxykynurenamine
4-Hydroxy-L-tryptophan
L-Kynurenine
Leucine
L-Norleucine
Isoxanthopterin
78-Dihydroxanthopterin
L-Phenylalanine
D-Phenylalanine
Pyrimidodiazepine
2-Aminomuconate
5-Hydroxykynurenine
